# In silico spectral libraries by deep learning facilitate data-independent acquisition proteomics

**DOI:** 10.1038/s41467-019-13866-z

**Published:** 2020-01-09

**Authors:** Yi Yang, Xiaohui Liu, Chengpin Shen, Yu Lin, Pengyuan Yang, Liang Qiao

**Affiliations:** 1 Department of Chemistry, Shanghai Stomatological Hospital, and Institutes of Biomedical Sciences, Fudan University, Shanghai, 200000 China; 2 Shanghai Omicsolution Co., Ltd., Shanghai, 200000 China; 3 College of Engineering and Computer Science, The Australian National University, Canberra, ACT 0200 Australia

**Keywords:** Proteomics, Proteomic analysis, Data mining, Proteome informatics

## Abstract

Data-independent acquisition (DIA) is an emerging technology for quantitative proteomic analysis of large cohorts of samples. However, sample-specific spectral libraries built by data-dependent acquisition (DDA) experiments are required prior to DIA analysis, which is time-consuming and limits the identification/quantification by DIA to the peptides identified by DDA. Herein, we propose DeepDIA, a deep learning-based approach to generate in silico spectral libraries for DIA analysis. We demonstrate that the quality of in silico libraries predicted by instrument-specific models using DeepDIA is comparable to that of experimental libraries, and outperforms libraries generated by global models. With peptide detectability prediction, in silico libraries can be built directly from protein sequence databases. We further illustrate that DeepDIA can break through the limitation of DDA on peptide/protein detection, and enhance DIA analysis on human serum samples compared to the state-of-the-art protocol using a DDA library. We expect this work expanding the toolbox for DIA proteomics.

## Abstract (web summary)

Data-independent acquisition (DIA) is an emerging technology in proteomics but it typically relies on spectral libraries built by data-dependent acquisition (DDA). Here, the authors use deep learning to generate in silico spectral libraries directly from protein sequences that enable more comprehensive DIA experiments than DDA-based libraries.

## Introduction

With the ability to identify and precisely quantify thousands of proteins from complex samples, liquid chromatography (LC)-tandem mass spectrometry (MS/MS) has been the most widely used tool for proteomic studies over the past decades^1,2^. Recent advances in the data-independent acquisition (DIA) technique allow systematic and unbiased proteomic measurement. In DIA experiments, the mass spectrometer performs a sequence of MS/MS scans within defined isolation windows in each acquisition cycle, recording fragmentation information of all peptides present in a sample^3^. Nevertheless, data analysis for DIA is extremely difficult since the fragments of various precursor ions can present on one MS/MS spectrum. For the past few years, a wide variety of strategies have been developed to analyze DIA data, including spectrum-centric and peptide-centric strategies^4^. Spectrum-centric workflows, such as DIA-Umpire^5^ and Group-DIA^6^, generate pseudo-MS/MS spectra for each precursor from DIA data for routine data-dependent acquisition (DDA) database search by assembling precursor-fragment groups based on the elution profiles of precursor and fragment ions. In peptide-centric methods, target peptides are queried against DIA data to extract the best candidate chromatogram signals using prebuilt spectral libraries, also known as peptide query parameters, or peptide assays, containing the information of retention time (RT) and fragment ions^7^. As an alternative, peptide query can also be applied to individual DIA MS/MS spectra by spectral matching tools, such as MSPLIT-DIA^8^. It has been reported that tools that rely on prior knowledge in the form of spectral libraries deal better with low selectivity data than library-free tools^9^, and peptide-centric approaches perform better to exploit highly comprehensive DIA data than spectrum-centric methods^10^. To date, a sample-specific spectral library, which is typically generated from DDA data acquired a priori from fractionated or enriched samples on the same instrument, is necessary in most studies using DIA. The method is not only time-consuming but also limits the identification/quantification by DIA to the peptides identified by DDA, which hinders the inherent advantages of DIA of unbiased measurement. In this regard, it is of great value to generate in silico spectral libraries containing predicted RT and fragment ions with quality comparable to that of experimental spectral libraries for DIA analysis.

A variety of RT prediction methods have been proposed, including look-up approaches, index-based methods, modeling-based methods, and machine learning-based methods^11^. Look-up approaches keep a table of previously observed RT for a set of peptides, where peptide standards are used for interconversion of RT and normalized RT (iRT) across different LC setups^12^. Modeling-based methods, such as BioLCCC^13^, predict RT based on structure of peptides and their interactions with LC columns using statistical physics. Index-based methods aim at estimating the contributions of each individual amino acid to peptide RT, which are often referred to retention coefficients, to form a retention index of peptide^14^. SSRCalc^15^ is currently the most widely used index-based tool, which has been integrated into the targeted proteomic tool, Skyline^16^, for RT scheduling. Machine learning-based methods use a set of peptides with known features and their RT to train a predefined model, such as artificial neural networks^17^ or support vector machines^18^. Nevertheless, machine learning-based methods rely on peptide feature selection, which is usually performed manually upon personal knowledge. As numerous factors are involved in peptide separation, the lack of suitable representations of peptide features, such as secondary structure^19^, leads to prediction errors. For peptide MS/MS spectrum prediction, there have also been tools developed, including kinetic model-based methods such as MassAnalyzer^20^ and MS-Simulator^21^, and machine learning-based methods like PeptideART^22^ and MS^2^PIP^23^. However, it has been shown that prediction performance of PeptideART across different experiments is significantly lower than those within the same experiment^24^. More powerful tools are required due to the complexity of peptide fragmentation and retention in LC.

Over the past years, deep learning has enabled many practical applications and attracted extensive attention. Deep neural networks, including convolutional neural network (CNN) and recurrent neural network (RNN), can learn different representations of objects automatically, recognizing complex patterns from large datasets^25^. Efforts have been made using deep neural networks for MS/MS spectrum prediction^26–28^, de novo peptide sequencing^29^ and RT prediction^27^, indicating great potential of deep learning in the field of proteomics.

**Fig. 1:**
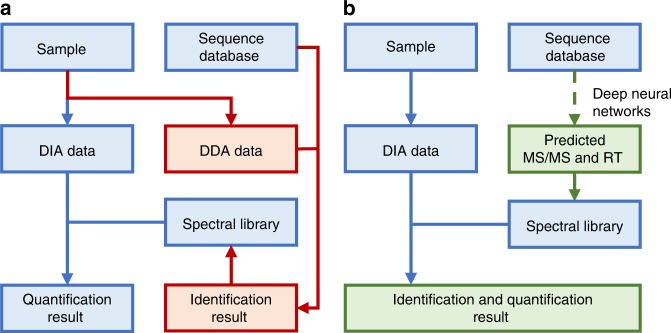
The workflow of conventional DIA analysis and DeepDIA. **a** In conventional DIA analysis, DIA target extraction is performed using sample-specific spectral libraries built with DDA result. **b** DeepDIA uses deep neural networks to generate in silico spectral libraries from protein or peptide sequence databases.

Herein, we present DeepDIA, a deep learning-based method to generate in silico spectral libraries to support DIA analysis (Fig. 1). In contrast to Prosit^27^, another recently reported tool that pursues a general deep learning model for MS/MS and RT prediction by taking collision energy (CE) into consideration, DeepDIA aims at training instrument-specific models for more accurate MS/MS spectrum and RT prediction. In addition, DeepDIA can select a list of target peptides to be included in in silico spectral libraries from protein sequence databases, e.g. SwissProt, by predicting the MS detectability of candidate proteotypic peptides. The in silico spectral libraries are readily applicable to data analysis using state-of-the-art DIA analysis software, e.g. Spectronaut^30^. We benchmark the performance of DeepDIA on datasets of HeLa cells and mixed proteome samples, and the results are comparable to those obtained with DDA-based sample-specific spectral libraries. Instrument-specific libraries by DeepDIA outperform Prosit in terms of detectable peptides and proteins as well as reproducibility among technical replicates.

We further demonstrate the applicability of DeepDIA on a dataset of human serum samples. Compared to the state-of-the-art protocol using the DDA-based library, the number of identified and quantified proteins is increased by >100% using an in silico spectral library wherein the peptide sequences are from human plasma/serum data of previous projects in our labs. Accuracy of the identification results is validated using a standard mixture containing >800 stable isotope labeled reference peptides from >500 proteins in human serum. We expect this method contributing to complete profiling of blood proteome samples across studies and laboratories. We have made the in silico spectral libraries as well as the DeepDIA tool freely available to expand the toolbox for DIA proteomics.

## Results

### Performance evaluation of peptide MS/MS and iRT prediction

**Fig. 2:**
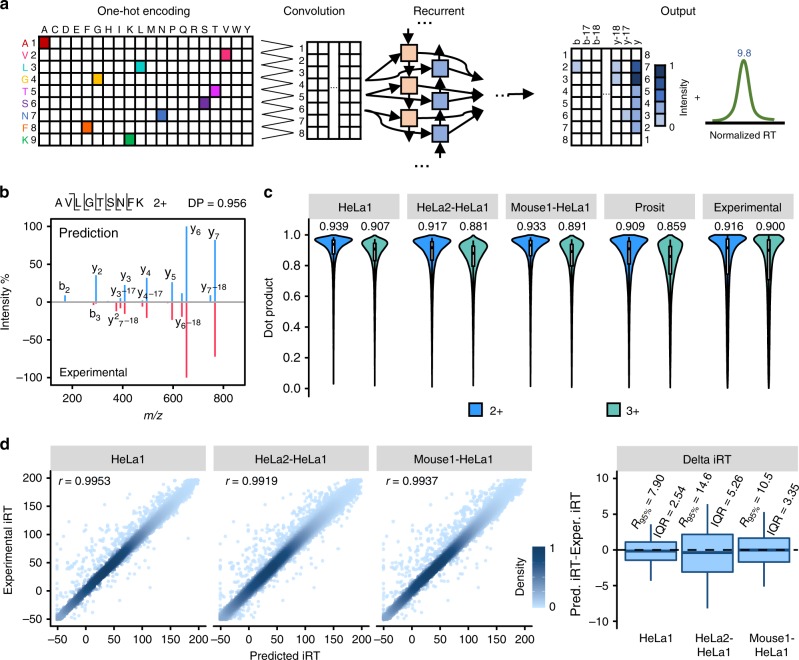
Peptide MS/MS spectrum and iRT prediction. **a** A graphical illustration of the deep neural networks for peptide MS/MS spectrum and iRT prediction. **b** A representative spectral match of a peptide (AVLGTSNFK 2+) between its higher energy collisional dissociation (HCD) MS/MS spectrum and the corresponding in silico predicted MS/MS spectrum. **c** The distributions of dot products computed between predicted and experimental b/y/neutral loss peak intensities. The medians are indicated. The boxes and whiskers show the quantiles and 95% percentiles, respectively. **d** Pearson correlation coefficients (*r*) and the differences computed between predicted and experimental normalized retention time (iRT). Color gradation indicates relative density of data points. The boxes show interquartile ranges (IQR), and the whiskers show 95% percentiles; no outliers are shown. 2+: doubly charged precursors; 3+: triply charged precursors. Source data are provided as a Source Data file.

In DeepDIA, MS/MS spectra and iRT of target peptides are predicted using a hybrid model that combines CNN and bi-directional long-term and short-term memory (BiLSTM, a widely used variant of RNN) network (Fig. 2a). The model takes a peptide sequence as an input, and outputs relative intensities of b/y product ions at each possible fragmentation site including neutral loss of ammonia or water, as well as iRT of the peptide. For details of the model architecture and training procedure, see Methods section.

For performance evaluation, we trained and validated the model with four DDA LC-MS/MS datasets of two organisms acquired on Q Exactive HF mass spectrometers in two laboratories (HeLa1, Mouse1, and Mouse2 from one lab, and HeLa2 from another lab; see Supplementary Table 1 for details)^31,32^. Each dataset was randomly partitioned into two subsets, where 2/3 were used for training and the remaining 1/3 for test. The distributions of dot products (DP)^33^ were computed between the predicted and experimental b/y/neutral loss peak intensities on the test dataset as presented in Fig. 2c. The median DP was as high as 0.939 for doubly charged precursors and 0.907 for triply charged precursors when the dataset used for model training and validation was from the same lab and the same organism (HeLa1 in Fig. 2c), better than those calculated by comparing different experimental spectra of the same precursors within a dataset (Experimental in Fig. 2c). For cross-organism validation (Mouse1-HeLa1 in Fig. 2c,) and cross-lab validation (HeLa2-HeLa1 in Fig. 2c), median DPs were 0.933 and 0.891, and 0.917 and 0.881, for doubly and triply charged precursors, respectively, wherein the models were trained using Mouse1 and HeLa2 data, respectively. Pearson correlation coefficients (*r*) of predicted and experimental iRT were higher than 0.99 and the interquartile ranges (IQR) of the differences between predicted and experimental iRT were smaller than 3 when the dataset used for model training and validation was from the same lab and the same organism (HeLa1 in Fig. 2d). IQRs were 3.35 and 5.26 for cross-organism (Mouse1-HeLa1 in Fig. 2d) and cross-lab validation (HeLa2-HeLa1 in Fig. 2d), wherein the models were trained using Mouse1 and HeLa2 data, respectively.

We computed DPs of experimental b/y peak intensities of the same precursors across several HeLa datasets from different labs^31,32,34,35^ (Supplementary Fig. 1a and Supplementary Note 1). Similarities of MS/MS spectra were low across different types of Orbitrap mass spectrometers. For the same type of mass spectrometers in different labs, similarities of MS/MS spectra were still a bit lower than experimental repeats in the same lab, possibly due to differences in instrumental settings and instrumental status between labs. We also compared experimental iRT of the same peptides in different HeLa datasets (Supplementary Fig. 2a).

The results showed that prediction using models trained with data from the same organism and the same lab were better than experimental repeats. The change of lab gave higher impact on the accuracy of prediction than the change of organism. For data from different organisms but the same lab, the prediction results were still comparable to experimental repeats within a lab and better than cross-lab experimental repeats. On the HeLa1 data, the performance of MS/MS prediction by DeepDIA with models trained by HeLa1 and Mouse1 were very close, indicating that good cross-sample prediction is feasible when keeping the instrument same.

We compared the performance of DeepDIA on peptide MS/MS and RT prediction to Prosit^27^, and other existing tools (Supplementary Note 2 and 3). Different normalized CE parameters were tested for Prosit on HeLa1 data (Supplementary Fig. 1b), and performance of MS/MS prediction by Prosit with the optimal CE was still worse than DeepDIA with models trained by HeLa1 (same-organism and same-lab), HeLa2 (same-organism and cross-lab), and Mouse1 (cross-organism and same-lab) (Fig. 2c). Test on Mouse1 data came to similar results (Supplementary Fig. 1c). We further compared DeepDIA, Prosit, pDeep^26^, and MS^2^PIP^23^ on HeLa2 data. The performance of DeepDIA was similar to pDeep wherein both models were trained with Mouse2 data (cross-organism and cross-lab), slightly worse than Prosit for 2+ precursors, and better than Prosit for 3+ precursors (Supplementary Fig. 1d). All the deep learning-base methods outperformed MS^2^PIP. For RT prediction, DeepDIA, Prosit and SSRCalc^15^ were compared on HeLa2 data, and DeepDIA trained with Mouse2 data (cross-lab and cross-organism) outperformed the latter two tools (Supplementary Fig. 2b-2d). From the results, the performance of DeepDIA trained with remote data in MS/MS prediction was similar to Prosit that was also trained with remote data. When DeepDIA was trained with data from the same organism or the same instrument, especially the same instrument, it outperformed Prosit in MS/MS and RT prediction.

### Benchmarking DeepDIA on HeLa and mixed proteome datasets

**Fig. 3:**
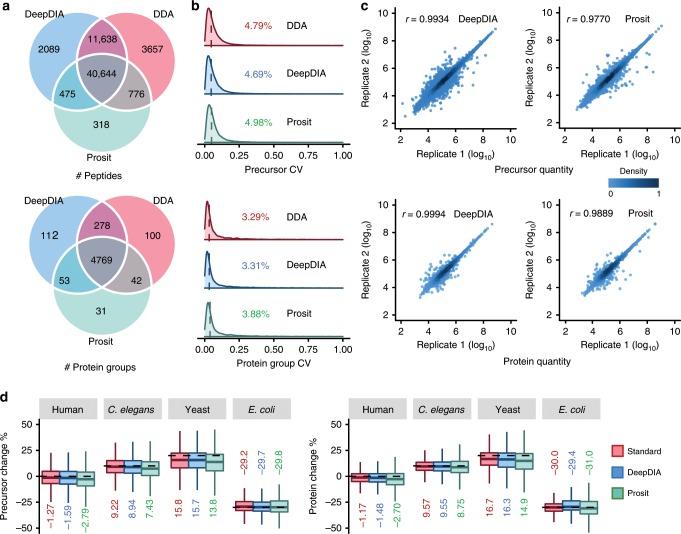
Performance comparison of predicted libraries and DDA libraries for DIA analysis. **a** The numbers of peptides and protein groups detected from a dataset of HeLa cells (HeLa1) using HeLaDDA (DDA), HeLaPredicted (DeepDIA), and HeLaProsit (Prosit) libraries. The Prosit library was built with the optimal normalized collision energy (CE = 30). Among the 3657 peptides only detected using HeLaDDA, 1886 with 1+ or >3+ charge state were absent in the predicted libraries (HeLaPredicted and HeLaProsit). Among the peptides detected using HeLaDDA and HeLaPredicted, 674 (639 using HeLaDDA and 376 using HeLaPredicted) with length > 30 amino acids were absent in the HeLaProsit library. **b** The distributions of coefficient of variation (CV) of peptide precursor and protein group quantification results detected in three technical replicates of HeLa1 using the three libraries. The medians are indicated. **c** Pearson correlation coefficients (*r*) between replicates of precursor and protein group quantification results of HeLa1. Color gradation indicates relative density of data points. **d** Box plot visualization of percent change of identified precursors and proteins of two mixed proteome samples. Percent changes were calculated based on the mean quantities in three replicates of each sample. Only overlapping identifications by the three libraries (Standard: MixStandard, DeepDIA: MixPredicted, Prosit: MixProsit) are shown. The medians are indicated. The boxes indicate the interquartile ranges (IQR), and whiskers indicate 1.5 × IQR values; no outliers are shown. Details of the spectral libraries are described in Supplementary Table 2. The dashed lines indicate theoretical fold changes of the organisms. Source data are provided as a Source Data file.

For benchmark purposes, we performed DIA analysis using Spectronaut on a dataset of HeLa cells containing three DIA technical replicates acquired on a Q Exactive HF mass spectrometer^31^ (HeLa1, see Supplementary Table 1 for details), using a sample-specific spectral library built with DDA experiments (HeLaDDA), an in silico library predicted by DeepDIA (trained with HeLa1 data) containing the doubly and triply charged precursors from the DDA results (HeLaPredicted), and in silico libraries containing the same precursors as HeLaPredicted and predicted by Prosit with different normalized CEs, respectively (see Supplementary Table 2 and Supplementary Note 4 for details). Precursor and protein group level *Q*-value was set to 0.01. More details are described in Methods section. The detected peptides as well as protein groups are listed in Supplementary Data 1 and statistics of the results are shown in Fig. 3a–c. At peptide level, 54,846 peptides were identified using the HeLaPredicted library, and 52,282 of them were shared with those identified using the HeLaDDA library. Among the 3657 peptides identified only by the HeLaDDA library, 1886 with single or more than triple charges were absent in the HeLaPredicted library. At protein group level, 5212 and 5189 protein groups were identified using the HeLaPredicted library and HeLaDDA library, respectively, with 5047 shared by the two methods. Results of Prosit predicted libraries with different normalized CEs are presented in Supplementary Fig. 3a and 3b. With the optimal CE, 42,213 peptides and 4895 protein groups were identified. Coefficients of variation (CVs) of precursors and protein group quantification results were calculated among the three technical replicates as shown in Fig. 3b. The median CVs were lower than 5 and 4% using the three libraries at precursor and protein group level, respectively. The median CVs using the HeLaPredicted library were comparable to those using the HeLaDDA library, and smaller than those using the Prosit library. Pearson correlation coefficients (*r*) of quantification results between replicates using the HeLaPredicted library was higher than those using the Prosit library at both precursor and protein group level (Fig. 3c). Apex RTs of precursors detected using the predicted libraries by DeepDIA were highly consistent with those using the HeLaDDA library (Supplementary Fig. 3c).

DIA analysis of HeLa1 was also performed using the Pan-Human library built on Q-TOF covering >10,000 human proteins^36^, and an in silico library (PanPredicted, with DeepDIA model trained using HeLa1 data) containing the doubly and triply charged precursors without variable modifications in the Pan library, respectively (Supplementary Table 2, Supplementary Fig. 4 and Supplementary Data 2). There were 43,005 peptides and 4720 protein groups identified by using both libraries. Extra 3256 peptides and 109 protein groups were identified using the Pan library, while extra 10,253 peptides and 842 protein groups were identified using the PanPredicted library. Therefore, the instrument-specific model predicted library outperforms the large heterogeneous experimental library, i.e. Pan-Human.

The performance of DeepDIA was further evaluated on a dataset of mixed proteome samples containing peptides from *Homo sapiens*, *Caenorhabditis elegans*, *Saccharomyces cerevisiae* and *Escherichia coli* with different abundance (Sample 1: Sample 2 1:1 for *H. sapiens*, 1:1.1 for *C. elegans*, 1:1.2 for *S. cerevisiae*, and 1:0.7 for *E. coli*, see Supplementary Table 1 for details) acquired on a Q Exactive HF mass spectrometer^31^. DIA analysis was performed using a standard DDA library (MixStandard), an in silico library predicted by DeepDIA (MixPredicted) trained with HeLa1 data, and an in silico library predicted by Prosit with optimized CE (MixProsit), respectively (Supplementary Note 4, Supplementary Table 2, Supplementary Fig. 5 and Supplementary Data 3). Based on the mean quantities in three replicates of each sample, percent changes of detected precursors and protein groups of the two samples were calculated and visualized in Fig. 3d. Percent changes of *H. sapiens*, *C. elegans*, and *E. coli* were close to the theoretical values using the MixStandard, MixPredicted and MixProsit libraries. For *S. cerevisiae*, percent changes were underestimated by using all the three libraries. The results by using the MixPredicted library were comparable to those using the MixStandard library, and better than those using MixProsit library at both precursor and protein group level (Supplementary Fig. 5), indicating that DeepDIA trained with data from the same instrument but different samples outperformed Prosit in terms of generating in silico spectral libraries for DIA analysis.

### In silico spectral libraries of large size

**Fig. 4:**
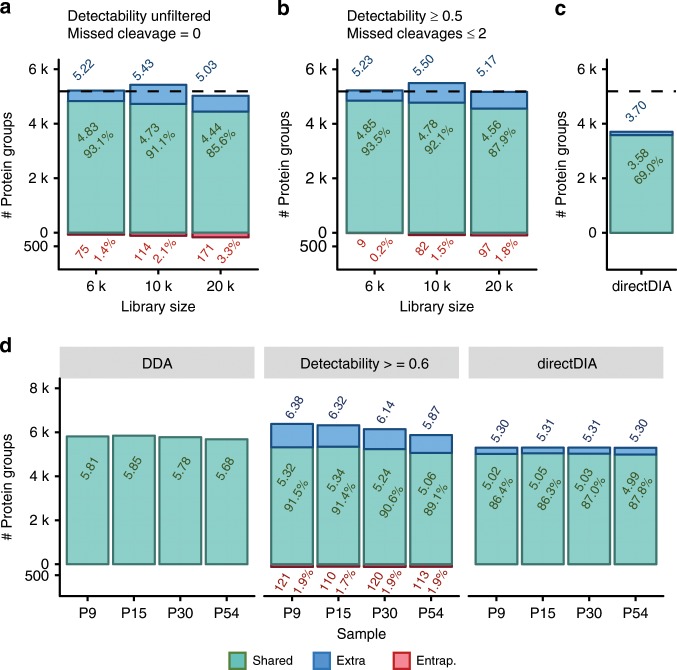
DIA analysis results using large in silico spectral libraries. **a** The numbers of protein groups detected from a dataset of HeLa cells (HeLa1) using HeLaProt (6 k proteins), PanProt (10 k proteins), and HumanProt (20 k proteins). The libraries include all in silico tryptic digested peptides without missed cleavage. **b** The numbers of protein groups detected from HeLa1 using HeLaProt50 (6 k proteins), PanProt50 (10 k proteins), and HumanProt50 (20 k proteins). The libraries include in silico tryptic digested peptides with ≤2 missed cleavages and a detectability score ≥0.5. **c** The numbers of protein groups detected from HeLa1 using directDIA. **d** The numbers of protein groups detected from a dataset of mouse tissue (Mouse1) using the DDA-based library, MouseProt60 (an in silico library generated from SwissProt *Mus musculus* database with a detectability score threshold of 0.6 and ≤2 missed cleavages), and directDIA. Details of the spectral libraries are described in Supplementary Table 2. The dashed lines indicate the numbers of protein groups detected using the DDA-based libraries. Overlapping identifications with the DDA-based libraries are referred as “shared”. Protein group numbers, sensitivities and entrapment (Entrap.) percentages are indicated.

Motivated by the results above, we explored the current limits of DeepDIA for direct analysis of DIA data without DDA analysis on the same sample. For this purpose, the performance of DeepDIA was tested using three large in silico spectral libraries, i.e. HeLaProt containing >6000 proteins identified by sample-specific DDA experiments, PanProt containing >10,000 proteins in HeLaProt or the Pan-Human library, and HumanProt containing >20,000 proteins from SwissProt *H. sapiens* database. Tryptic specific digested peptides without missed cleavage were in silico generated and considered by the DeepDIA, which was trained using the HeLa1 data. For large spectral libraries, there were a few differences in search condition (described in Methods section). An entrapment strategy^37^ was used to approximately estimate false positive identifications by adding proteins from other organisms to the libraries. In all the analyses, we kept the number of entrapment proteins similar to that of the organism specific proteins, and consequently there were 6173 (human) + 6622 (entrapment) proteins and 207,061 (human) + 155,776 (entrapment) peptides in HeLaProt, 10,639 (human) + 10,649 (entrapment) proteins and 358,849 (human) + 270,503 (entrapment) peptides in PanProt, and 20,163 (human) + 19,226 (entrapment) proteins and 585,934 (human) + 455,253 (entrapment) peptides in HumanProt (see Methods and Supplementary Table 2 for details). The DIA analyses results by the large in silico spectral libraries are presented in Fig. 4a, Supplementary Fig. 6a, 7a and 7b, and Supplementary Data 4. Although the same *Q*-value filter was applied on all the analyses, there were a small percentage of entrapment proteins remaining. As the size of spectral library increased, sensitivities (percentage of the number of proteins shared by using the HeLaDDA and predicted libraries to those identified using the HeLaDDA library) of identification at protein group levels declined from 93.1% to 85.6%, while entrapment percentages (percentage of the number of entrapment proteins to all of those identified using the predicted library) increased from 1.4% to 3.3%.

In order to build an in silico spectral library directly from a proteome database similar to the DDA spectral library, criteria should be established to select a list of target peptides from a protein to be included in the spectral library. We modified the model for RT prediction, and applied it to predict the detectability of peptides by mass spectrometry (Supplementary Note 5 and Supplementary Fig. 8a–c). A detectability prediction model was trained with a dataset of HeLa and HEK-293 cells (HeLa&HEK, Supplementary Table 1), which was from the lab acquiring the HeLa1 dataset. Then, we built another three spectral libraries (see Supplementary Table 2 for details), denoted as HeLaProt50 (6151 human proteins + 6459 entrapment proteins, 161,376 human peptides + 129,500 entrapment peptides), PanProt50 (10,591 human proteins + 10,458 entrapment proteins, 273,050 human peptides + 220,790 entrapment peptides) and HumanProt50 (19,841 human proteins + 18,909 entrapment proteins, 431,624 human peptides + 416,125 entrapment peptides), containing tryptic peptides with ≤2 missed cleavages and with detectability scores ≥ 0.5 from all the proteins (including entrapment entries) in HeLaProt, PanProt and HumanProt, respectively. The MS/MS and RT prediction models were trained using the HeLa1 data. Since there was no significant change on the protein number before and after filtering for both entrapment and human, we can use entrapment percentage to compare the error rates before and after filtering relatively. Using the three new libraries, entrapment percentages at protein group level were <2%, and sensitivities were higher than those using the in silico libraries without detectability filtering (Fig. 4b, Supplementary Fig. 6b, 7c and 7d, and Supplementary Data 4). Using the HeLaProt50 library, the entrapment percentage decreased to 0.2%, while the sensitivity was >93% at protein group level. Even with the HumanProt50 library, the number of detected protein groups was still approximately equal to that detected with the HeLaDDA library.

At peptide level, when detectability prediction was applied, the number of identified peptides by DeepDIA was larger than or comparable to that by using the DDA library, and significantly larger than that by DeepDIA without detectability prediction. The overlap of peptide identifications using the DDA library and the predicted library was lower than that of protein group identifications (Supplementary Fig. 6). It should be noticed that we chose the identification results by using the HeLaDDA library as reference for sensitivity calculation, which does not indicate that the proteins/peptides identified only by using the predicted libraries were wrong. Since the protein level overlap was high, most of the peptides only identified using the predicted libraries were from the protein groups also identified using the DDA library.

We further tested DeepDIA (detectability prediction trained with HeLa&HEK, MS/MS and RT prediction trained with HeLa1) with detectability prediction on a dataset of four groups (P9, P15, P30, and P54) of mouse tissue samples (Mouse1, see Supplementary Table 1 for details) for cross species validation. Different detectability score thresholds were set to build in silico libraries from SwissProt *Mus musculus* database (Supplementary Fig. 8d). Using the in silico library with a detectability score threshold of 0.6 and ≤2 missed cleavages (MouseProt60, Supplementary Table 2), entrapment percentages were <2% and sensitivities were ~90% at protein group level (Fig. 4d and Supplementary Data 5). The corresponding peptide level information is given in Supplementary Fig. 6d.

We have further adapted a two-step approach for DIA analysis using spectral libraries generated from proteome-scale databases. The library used for the second search on Mouse1 was generated from the first search results using MouseProt60 in silico spectral library (Supplementary Table 2). Proteins combined from four groups (P9, P15, P30, and P54) of samples detected in the first search were in silico digested (≤2 missed cleavages) and protein inference was re-performed based on the peptides after detectability filtering (detectability score ≥ 0.6). Consequently, the library contained 7424 proteins (7380 protein groups) from *M. musculus*, while 6424 proteins (6340 protein groups) from *S. cerevisiae* and 4297 proteins (4235 protein groups) from *E. coli* were added as entrapment. In the identification results of the second search, the entrapment percentages were ≤0.5%, and the sensitivities were ranging from 91.3 to 93%. More protein groups were identified for each sample than that using the DDA-based library (Supplementary Note 6 and Supplementary Fig. 9). Similar performance was also observed on the HeLa1 data (Supplementary Fig. 9). During the second search, the library size was smaller and the library was more specific to the sample, which could lead to better performance in peptide and protein identification.

For benchmark purposes, directDIA, a spectrum-centric library-free tool in Spectronaut, was performed on the HeLa1 and Mouse1 dataset with 1% precursor and protein group level *Q*-value. Sensitivities were 69% and ~87% at protein group level using directDIA for the HeLa1 and Mouse1, respectively (Fig. 4c, d). The numbers of identified proteins and peptides were significantly smaller than that by DeepDIA and the DDA libraries (Fig. 4, Supplementary Fig. 6, and Supplementary Fig. 8).

### Enhanced protein detection from human serum by DeepDIA

**Fig. 5:**
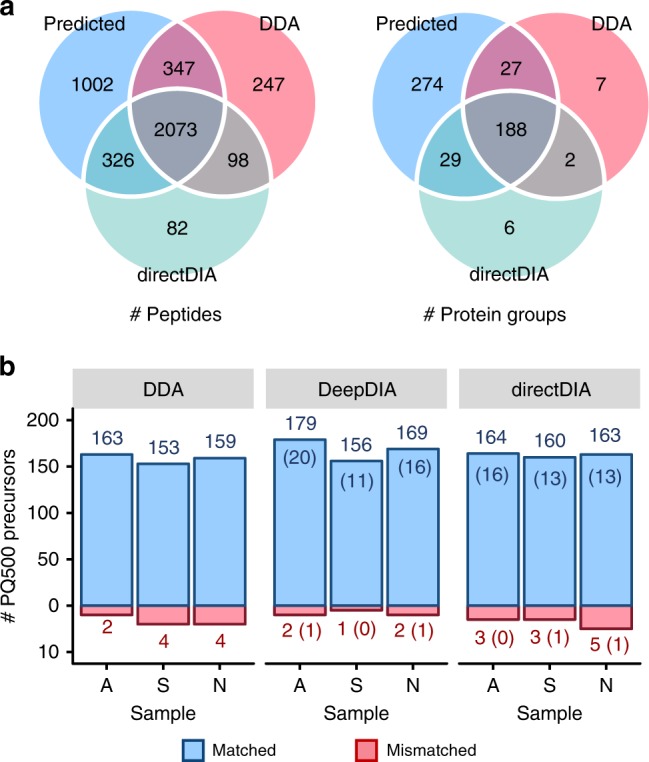
DIA analysis results of a dataset of human serum. **a** The numbers of peptides and protein groups detected using the predicted library (PlasmaPredicted), the DDA-based library (SerumDDA), and directDIA. **b** Numbers of PQ500 precursors without isotope label detected in the three samples using the DDA-base library (SerumDDA), the DeepDIA predicted library (PlasmaPredicted) and directDIA. Numbers in parentheses indicate the numbers of PQ500 precursors absent in the result using the DDA-based library. A, S, and N represent the three serum samples, respectively. Details of the spectral libraries are described in Supplementary Table 2.

Analysis of proteins from blood is an important clinical application of proteomics, but is challenged by the extreme dynamic range of protein abundance. From human plasma/serum data of previous projects in our labs, we collected a sequence database containing 27,142 peptides of 2543 protein groups, and built an in silico library (PlasmaPredicted, see Supplementary Table 2 for details, PXD014108) using DeepDIA. The PlasmaPredicted library was tested on a dataset of three human serum samples (A, S, N) acquired on a Q Exactive HF mass spectrometer with six DIA runs without high abundance protein (HAP) depletion. Also, a project-specific library of the serum samples (containing 7484 peptides of 877 protein groups) was built based on DDA with HAP depletion and pre-fractionation by high pH reverse phase (RP) LC. The DIA results are presented in Supplementary Data 6. With the in silico library, >400 protein groups were detected and quantified on average for each run (Supplementary Fig. 10a). From the accumulated results of all the runs, 3748 peptides and 518 protein groups were detected with the in silico library. Among them, 1328 peptides and 303 protein groups were missed using the DDA-based library (Fig. 5a). DeepDIA detected 36% more peptides and 131% more protein groups than the DDA-based approach, and 45% more peptides and 130% more protein groups than directDIA (Fig. 5a). Using the in silico library, peptide and protein detection was no longer limited by DDA results of the samples, and hence more peptides/proteins were detected from the DIA data.

PQ500 stable isotope labeled reference peptides were added to the samples in three runs. Using the in silico library, 181, 157, and 171 precursors were detected for the PQ500 peptides without isotope label, which were originally from the sample. Among them, 20, 11, and 16 were missed using the DDA-based library (Fig. 5b). By comparing the RT of the PQ500 precursors detected in each run using the in silico library with the corresponding isotope labeled peptides from the kit using the official PQ500 library, 179, 156, and 169 precursors were matched with an absolute RT difference of <0.5 min. In each run, ~110 protein groups containing these matched PQ500 peptides were detected, and distributed throughout the intensity range of detected protein groups (Supplementary Fig. 10b). Considering the “matched” precursors as correct identification, the accuracies of detection using the in silico library were estimated as ~99% on average, higher than those using the DDA-based library and directDIA.

## Discussion

DIA technique has enabled fast quantitative analysis of large cohorts of samples. Currently, spectral library generation in DIA quantitative proteomic experiments requires sample pre-fractionation and DDA experiments, which is time-consuming and limits the detection to peptides identified by DDA. Using deep learning, we generate in silico spectral libraries with accurate prediction of fragment intensities, iRT and peptide detectability by MS, and demonstrate that the performance of predicted libraries is comparable to DDA-based libraries.

Reproducibility of peptide fragmentation and retention is influenced by types of instruments and changes of instrumental settings, e.g. LC column, LC flow rate, electrospray voltage, ion optics, scan range, resolution, AGC target, collision energy, etc., between labs. It has been reported that experimental factors do influence iRT precision, and it is recommended to perform library generation and analytical measurements on the same system^38^. On the analysis of the HeLa dataset and the mixed proteome dataset, we trained deep neural networks using the DDA data provided by the lab generating the DIA data, and achieved better performance than Prosit that was trained using the reference MS/MS data from the ProteomeTools project. Since it is not feasible to consider all the instrumental settings during the training of deep neural networks, we suggest generating lab and instrument-specific models for DIA analysis, instead of developing a global model.

In this study, in silico spectral libraries were generated in two ways, i.e. from peptide lists and from protein sequence databases. From peptide lists in public libraries, e.g. Pan-Human, instrument-specific libraries were built for DIA analysis, achieving better results than the original public libraries. We expect that scientists can take full advantage of community resources using models trained with data acquired on their own instruments to improve their DIA analysis. In silico spectral libraries can also be built from protein sequences in public database, e.g. SwissProt, for direct analysis of DIA data without peptide-level prior knowledge. However, a current challenge of DIA analysis using proteome-scale libraries is the large query space. The increasing numbers of peptides queried favor the occurrence of false positives and compromise detection sensitivity. It has been pointed out that further investigations are required to establish appropriate strategies for statistical control of error rates in large-scale DIA studies, and to optimize strategies for reducing the query space in different applications^39^. We developed deep neural networks for peptide detectability prediction, enabling the selection of target peptides from proteins by setting a threshold of detectability score. More attempts, e.g. two-step search, have been taken to reduce the query space (Supplementary Note 6), and we anticipate that the issues of large query space can be overcome in the future. Also, instead of querying all the peptides in a spectral library, researchers can focus on a subset of proteins of interest for their specific biological questions^10^.

Plasma/serum proteomics holds great promise for the discovery of protein biomarkers for a range of diseases, such as early stage cancers^40^ and cardiovascular diseases^41^. However, the study of plasma proteome is challenged by the extreme dynamic range of protein abundance, i.e. over 12 orders of magnitude^42^. Efforts are being made on human plasma/serum proteome by many labs around the world. Based on data from our labs, an in silico plasma/serum proteome library was built using DeepDIA. With the in silico library, >400 protein groups were detected on average in a single DIA run without HAP depletion, which was double of those detected using state-of-the-art DDA-based library from the same data. By spike-in approach with stable isotope labeled reference peptides, the error rates of detection using the in silico library were estimated as low as those using the DDA-based library. The results indicate that DeepDIA coupled with state-of-the-art peptide-centric tools can break through the limitation of DDA on peptide/protein detection, and outperforms spectrum-centric approaches in proteomic study of blood sample. We have made the in silico plasma/serum spectral library as well as the DeepDIA tool freely available, and hope that they will contribute to complete profiling of blood proteome samples across studies and laboratories.

Although it is demonstrated here in the context of DIA proteomics, DeepDIA should also benefit any method that relies on spectral libraries or other prior knowledge, for instance targeted proteomics. MS/MS and RT prediction are complementary to shotgun experiments to extend targeted assays for selected reaction monitoring (SRM) or parallel reaction monitoring (PRM) experiments. Recently, a “global targeting” approach was proposed by unifying shotgun and targeted proteomics, extending the targeting concept to a proteome-wide scale^43^, and we expect it will also profit from in silico target lists generation.

In summary, we have demonstrated that instrument-specific models can outperform approaches like Prosit and pDeep in the generation of in silico spectral libraries for DIA data analysis, and that the in silico spectral libraries generated by instrument-specific models from public protein sequence database, e.g. SwissProt, are comparable to sample specific DDA-based libraries in the analysis of DIA data. We expect in the future that labs would train their instrument-specific DeepDIA models using DDA data acquired from fractionated peptides of a cell line, e.g. HeLa, and then use the models to analyze their DIA data of other samples without performing DDA experiments until significant changes in equipment performance are observed.

## Methods

### Sample preparation

Human serum samples were collected from three volunteers under the consent of the donors. For DDA analysis, high-abundant proteins (HAP) were depleted using Pierce Top 12 Abundant Protein Depletion Spin Columns (ThermoFisher Scientific, Rockford, USA) and Seppro IgY14 Spin Columns (Sigma-Aldrich, St. Louis, MO, USA). After that, 10 μL of samples were diluted 1:40 with urea buffer (8 M urea, 1% sodium dodecyl sulfate), exposed on ice for 30 min with vortex mixing every 10 min, and centrifuged at 12,000*g* for 20 min at 4 °C. The supernatant was collected, and proteins were quantified using Pierce BCA Protein Assay Kit (ThermoFisher Scientific, Rockford, USA).

For each sample, 100 μg of protein extracts were resuspended in 8 M urea at 1 mg mL^−1^. After adding 2 μL of 0.5 M tris(2-carboxyethyl)phosphine (TCEP), the sample was incubated at 37 °C for 1 h. Then 4 μL of 1 M iodoacetamide was added to the sample and the incubation was last for 40 min in dark at room temperature. After that, five volumes of −20 °C pre-chilled acetone was added to precipitate the proteins overnight at −20 °C. The precipitates were washed by 1 mL pre-chilled 90% acetone aqueous solution twice and then re-dissolved in 100 μL 100 mM tetraethylammonium tetrahydroborate (TEAB). Sequencing grade modified trypsin (Promega, Madison, WI, USA) was added at the weight ratio of 1:50 (enzyme:protein) to digest the proteins at 37 °C overnight. Peptides from each sample were purified and concentrated using Pierced C18 spin columns (ThermoFisher Scientific, Rockford, USA), and then quantified using Pierce Quantitative Colorimetric Peptide Assay (ThermoFisher Scientific, Rockford, USA).

### LC-MS/MS analysis

DDA was used to build a spectra library for DIA analysis. The peptides from each sample were pooled together and re-dissolved in buffer A (20 mM ammonium formate in water, pH 10.0, adjusted with ammonium hydroxide), and then fractionated by high pH reverse phase (RP) LC separation using an Ultimate 3000 system (ThermoFisher scientific, MA, USA) connected to an XBridge C18 column (4.6 mm × 250 mm, 5 μm) (Waters Corporation, MA, USA). High pH RPLC separation was performed using a linear gradient, starting from 5 to 45% B in 40 min (B: 20 mM ammonium formate in 80% acetonitrile, pH 10.0, adjusted with ammonium hydroxide). The column was re-equilibrated at the initial condition for 15 min. The column flow rate was maintained at 1 mL min^−1^ and the column temperature was maintained at 30 °C. Ten fractions were collected (4 min each). Each fraction was dried in a vacuum concentrator. The peptides were re-dissolved in solvent C (0.1% formic acid in water) and analyzed by an on-line nanospray Q Exactive HF mass spectrometer coupled with an EASY-nLC 1200 system (ThermoFisher Scientific, MA, USA). For each sample, 3 μL (1 μg) was loaded to an Acclaim PepMap C18 column (75 μm × 25 cm) (Thermo Fisher Scientific, MA, USA) and separated with a 120 min gradient, from 5 to 35% solvent D (0.1% formic acid in acetonitrile). The column flow rate was maintained at 200 nL min^−1^. The electrospray voltage of 2 kV versus the inlet of the mass spectrometer was used. The MS scan was performed with the following parameters: scan range (*m/z*) = 350 − 1600; resolution = 60,000; automatic gain control (AGC) target = 3e6; maximum injection time = 50 ms; dynamic exclusion = 30 s. The HCD MS/MS scan was performed with the following parameters: resolution = 15,000; AGC target = 5e5; maximum injection time = 60 ms; collision energy = 30.

Three serum samples from three individuals without HAP depletion were analyzed in DIA mode with two replicate runs for each sample. The peptides were re-dissolved in 30 μL solvent C and spiked with iRT Kit (Biognosys AG, Schlieren, Switzerland). For the second replicate run of each sample, PQ500 Reference Peptides Kit (Biognosys AG, Schlieren, Switzerland) was added. 3 μL (1 μg) of each sample was subjected to LC-MS/MS analysis. The MS scan was performed with the following parameters: scan range (*m/z*) = 350–1650; resolution = 60,000; AGC target = 3e6; maximum injection time = 20 ms. The HCD MS/MS scan was performed with the following parameters: resolution = 30,000; AGC target = 1e6; collision energy = 27; stepped collision energy = 5%. DIA was performed with 42 variable isolation windows with 1 Da overlap, and the total cycle time was 3 s. The other MS parameters as well as LC gradient conditions and LC column were the same as those in DDA.

### Database searching of DDA data

The DDA data were analyzed with SpectroMine (version 1.0.21621, Biognosys AG, Schlieren, Switzerland) assuming Trypsin/P as the digestion enzyme with maximum missed cleavages set to 2 and peptide length range set from 7 to 50. Carbamidomethyl (C) was specified as the fixed modification, and no variable modifications were specified. The HeLa, HEK293 and serum DDA data were searched against the SwissProt *Homo sapiens* database (access date 2018-04, 20,301 entries) downloaded from UniProt. The mouse data were searched against the SwissProt *Mus musculus* database (access date 2019-02, 17,006 entries). *Q*-value cutoff on precursor and protein level was applied 1%. Other parameters were default values.

### Training and validation of the deep neural networks

HCD MS/MS spectra of peptide precursors were collected from the HeLa1 (17 runs), HeLa2 (12 runs), Mouse1 (23 runs), and Mouse2 (15 runs) DDA data (Supplementary Table 1). For each peptide precursor, only one peptide spectrum match (PSM) with the minimum *Q*-value was kept. Singly charged peptides (6206 in HeLa1, 1278 in HeLa2, 9688 in Mouse1, and 26,700 in Mouse2) and peptides with charge states higher than 3+ (5683 in HeLa1, 11,827 in HeLa2, 10,105 in Mouse1, and 22,695 in Mouse2) were excluded because the amount of these precursors was too small for training and testing. As a result, PSMs of 69,577 peptides (57,198 doubly charged precursors and 27,468 triply charged precursors), 88,462 peptides (67,053 doubly charged precursors and 37,480 triply charged precursors), 72,282 peptides (50,792 doubly charged precursors and 27,584 triply charged precursors), and 171,832 peptides (132,854 doubly charged precursors and 76,588 triply charged precursors) were obtained from HeLa1, HeLa2, Mouse1, and Mouse2, respectively. Peaks of singly charged and doubly charged b/y product ions were extracted, as well as corresponding neutral loss (loss of ammonia or water) peaks.

A hybrid model based on CNN^44^ and BiLSTM networks^45^ were constructed for MS/MS spectrum prediction. The model takes a peptide sequence as input and converts it by one-hot encoding to a bit matrix. In the matrix, each row represents a kind of amino acid residue (20 rows in total), while each column stands for a residue position. The maximum sequence length is 50, and zero vectors are padded to peptides with length < 50 for the empty positions. Then a convolution layer (with 64 filters of size 2) scans across the input matrix to extract features from adjacent amino acid residues. A BiLSTM layer (128-dimensional) is used to model the sequential patterns of each cleavage position. A dropout layer (with rate of 0.5) is added to the model to avoid over fitting^46^. Through a dense layer (12 dimensional) with activation function of rectified linear units (ReLU)^47^, the model finally outputs an intensity matrix with each row for a type of product ion (b and y, including loss of water and ammonia, with charge states of 1+ and 2+, 12 rows in total) and each column for a cleavage site (49 columns in total). The model was compiled with loss function of mean square error (MSE) and the optimizer of adaptive moment estimation (Adam)^48^. Models for 2+ and 3+ precursors were trained separately. Each dataset was randomly partitioned into two subsets, where 2/3 were used for training and the remaining 1/3 for validation. Dot product (DP)^33^ was calculated between the predicted and experimental peak intensities.

The model for RT prediction was very similar to that for MS/MS spectrum prediction, except that kernel size of the convolution layer was set to 5 and the dimension of the dense layer was changed to output scalar values, i.e. normalized RT. The model to predict the detectability of peptides by mass spectrometry (Supplementary Fig. 8a) was similar to that for RT prediction, except slight modifications that the input sequence included not only the peptide, but also seven amino acids (pad with a blank if no amino acid is at the position) before and after the N-terminal and C-terminal cleavage sites, respectively, separated by a dot, e.g. “___MASK.LLRAVILGPPGSGK.GTVCQRI”, and thus the dimension of input layer is 22 (20 + 2). More details on the model to predict the detectability of peptides by mass spectrometry can be found in the Supplementary Note 5.

The models were implemented in Python (Anaconda distribution version 4.2.0) using Keras (version 2.2.4) with TensorFlow (version 1.11.0) backend. Data preprocessing and visualization were conducted with R (version 3.5.1). Running time for model training is described in Supplementary Note 7.

### Spectral library generation

Spectral libraries were generated from DDA search archives using Spectronaut (version 12.0.20491 and 13.3.190726, Biognosys AG, Schlieren, Switzerland) with default settings. For the generation of in silico spectral libraries, a list of sequences of target peptides, collected from DDA results or protein sequences by in silico digestion, was input to the deep neural networks for prediction of fragment intensities and iRT, which was written to a comma-separated values (CSV) file, and then imported to Spectronaut with default settings. Protein sequences were digested using Protein Digestion Simulator (version 2.2.6794). For in silico libraries without detectability filtering, Trypsin and Trypsin/P were set as the digestion enzyme with no missed cleavages, respectively, and the results were combined. For libraries with detectability filtering, Trypsin/P was set as the digestion enzyme with missed cleavages ≤2. Only peptides with length from 7 to 50 amino acids with mass ≤ 6000 Da were kept.

### DIA data analysis

Raw data of DIA were processed and analyzed by Spectronaut. Retention time prediction type was set to dynamic iRT. Data extraction was determined by Spectronaut based on the extensive mass calibration. Decoy generation was set to mutated. Interference correction on MS2 level was enabled. Peptide and protein level *Q*-value cutoff was set to 1%. For mixed proteome samples, SwissProt *H. sapiens* isoform database (access date 2018-06, 42,356 entries), UniProt Proteome *C. elegans* isoform database (access date 2019-03, 28,302 entries), SwissProt *S. cerevisiae* (strain ATCC 204508 / S288c) database (access date 2019-03, 6,721 entries) and SwissProt *E. coli* (strain K12) database (access date 2019-03, 4,480 entries) were used as protein sequence databases. For other datasets, protein database was set the same as those used in DDA searching.

For large spectral libraries, machine learning was performed across experiments, and protein groups with single hit (i.e. only one stripped peptide sequence) in each run were excluded. An entrapment strategy^37^ was used to compare false positive identification rates under the given *Q*-value. An entrapment library was built using proteins from other organisms with roughly equivalent size to the organism specific library (see Supplementary Table 2 for details). The organism specific library and the entrapment library were merged and used as the target library. Identification results were filtered by 1% *Q*-value by a target-decoy approach implemented by Spectronaut. The generation of decoy was on the whole target library including entrapment. As we introduced the entrapment entries in the target database, the entrapment hits in filtered target hits were considered as false positive results. Thus, we used entrapment percentage (percentage of the number of entrapment hits to the target hits) to compare the false positive rates relatively. It should be noted that the true error rate is higher than the entrapment percentage.

Peptide and protein reports were exported as CSV files, and subsequent statistic and visualization were performed with R scripts.

### Reporting Summary

Further information on research design is available in the Nature Research Reporting Summary linked to this Article.

## Supplementary information

Supplementary Information

Description of Additional Supplementary Files

Supplementary Data 1

Supplementary Data 2

Supplementary Data 3

Supplementary Data 4

Supplementary Data 5

Supplementary Data 6

Reporting Summary

## Data Availability

All raw mass spectrometry data, spectral libraries and search results are publicly available at the ProteomeXchange Consortium. Raw data of HeLa, HEK-293, mouse and mixed proteome samples are available with the dataset identifier PXD005573, PXD006932, PXD004452, and PXD009875 (see Supplementary Table 1 for details). All the models for MS/MS, RT and detectability prediction, the data used for model training to generate PlasmaPredicted (see Supplementary Table 2 for details), raw data of serum samples, all in silico spectral libraries and the saved projects from Spectronaut have been deposited to ProteomeXchange via the iProX^49^ partner repository with the dataset identifiers PXD014108 and IPX0001628000. The source data underlying Figs. 2c-d and 3b-d, as well as Supplementary Fig. 1, 2, 3b-c, 4b-c, 5b-c, 7, 8c and 10b are provided as a Source Data file. All other data are available from the corresponding author on reasonable request.

## Source data

Source Data

## Code availability

DeepDIA is open source and freely available on GitHub.
