# Supplementary material for: In silico spectral libraries by deep learning facilitate data-independent acquisition proteomics
Source: Nat Commun. 2020 Jan 9;11:146. doi: 10.1038/s41467-019-13866-z (PMC6952453; doi:10.1038/s41467-019-13866-z)
Supplement: Supplementary file 1 — Supplementary Information [file 41467_2019_13866_MOESM1_ESM.pdf]

In silico spectral libraries by deep learning facilitate data-independent acquisition proteomics

Yang et al.

Supplementary Table 1. LC−MS/MS datasets used in this study.

Name	Instrument	Description	Accession
HeLa1	Q Exactive HF (Reiter's lab)	HeLa cells. DDA without or with fractionation (17 runs), and 3 DIA technical replicates.<br>Raw file names:<br>C_D160304_S251-Hela-2ug-2h_MSG<br>C_D160331_S209-HPRP-HeLa-*_MSG<br>C_D160401_S209-HPRP-HeLa-*_MSG<br>Fig1_MP-DIA-120min120kMS1-22W30k-8dppp_MHRM	PXD005573
HeLa2	Q Exactive HF (Olsen's lab)	HeLa cells. DDA with fractionation (12 runs), and without fractionation with different LC gradients (15 min, 30 min and 60 min, 3 replicates for each condition).<br>Raw file names:<br>20161214_HF_DBJ_SA_Exp7b_Hela_100ug_12frac_30min<br>20161214_HF_DBJ_SA_Exp3B_Hela_1ug_15min_15000<br>20161214_HF_DBJ_SA_Exp3B_Hela_1ug_30min_15000<br>20161214_HF_DBJ_SA_Exp3B_Hela_1ug_60min_15000	PXD006932
Mouse1	Q Exactive HF (Reiter's lab)	Mouse barrel cortex. DDA with or without fractionation (23 runs), and DIA of samples of 4 groups with 3 replicates for each group.<br>Raw file names:<br>C_D160415_S209-BC-1m-HPRP-*_MSG<br>var_D150908_DDA_R01_S209-120min-22W-BC<br>var_D150908_DDA_R01_S209-2ul-BC-*-DDA<br>Fig5_MP-DIA-barrel-cortex-*-1_MHRM	PXD005573
Mouse2	Q Exactive HF (Reiter's lab, on an instrument different from Mouse1)	Mouse cerebellum. DDA with fractionation (15 runs).<br>Raw file names:<br>E_D170628_S450-HPRP-Cer-15-*_MSG	PXD005573
Mix	Q Exactive HF (Reiter's lab)	Mixed proteome samples containing *H. sapiens*, *C. elegans*, *S. cerevisiae*, and *E. coli* of differential abundance (Sample 1: Sample 2 1:1 for *H. sapiens*, 1:1.1 for *C. elegans*, 1:1.2 for *S. cerevisiae*, and 1:0.7 for *E. coli*) with 3 DIA technical replicates for each sample.<br>Raw file names:	PXD005573

		Fig3_MP-DIA-multi-organism-Run-S1_MHRM<br>Fig3_MP-DIA-multi-organism-Run-S2_MHRM	
HeLa&<br>HEK	Q Exactive HF<br>(Reiter's lab)	HeLa and HEK-293 cells. DDA without or with fractionation (20 runs).<br>Raw file names:<br>Fig4_HEK293-1m_DDA<br>Fig4_HEK293-1m-HPRP-*_DDA<br>Fig4_HeLa-1m _DDA<br>Fig4_HeLa-1m-HPRP-*_DDA	PXD005573
Serum	Q Exactive HF<br>(Our lab)	Three human serum samples without high-abundant proteins (HAP) depletion, each with 2 DIA runs without or with PQ500 Reference Peptides Kit.	PXD014108

Supplementary Table 2. Spectral libraries used in this study.

Name	Description
HeLaDDA	Generated from HeLa1 DDA search results.<br>96,354 precursors, 69,515 peptides, 6,180 (6,088) protein (groups).
HeLaPredicted[a]	Generated from precursors in HeLaDDA. Predicted using the model trained with HeLa1.<br>84,556 precursors, 67,513 peptides, 6,146 (6,054) protein (groups).
HeLaProsit[b]	Generated from precursors in HeLaDDA. Predicted using Prosit.<br>CE28: 83,648 precursors, 66,714 peptides, 6,137 (6,044) protein (groups).<br>CE30: 83,778 precursors, 66,810 peptides, 6,139 (6,046) protein (groups).<br>CE32: 83,791 precursors, 66,816 peptides, 6,139 (6,046) protein (groups).<br>CE34: 83,742 precursors, 66,800 peptides, 6,139 (6,046) protein (groups).<br>CE36: 83,557 precursors, 66,689 peptides, 6,139 (6,046) protein (groups).
Pan	The public Pan-Human library by Rosenberger *et al.* This library was built from data acquired on TripleTOF 5600+.<br>210,921 precursors, 149,066 peptides, 10,643 (10,456) protein (groups).
PanPredicted[a]	Generated from precursors in Pan. Predicted using the model trained with HeLa1.<br>166,943 precursors, 135,823 peptides, 10,465 (10,330) protein (groups).
MixStandard	Merged libraries of HeLa, HEK-293, *Caenorhabditis elegans*, *Saccharomyces cerevisiae*, and *Escherichia coli*. This library was built from DDA data acquired on Q Exactive HF.<br>*C. elegans*: 20,019 precursors, 17,780 peptides, 3,448 (2,662) protein (groups).<br>*E. coli*: 16,301 precursors, 13,074 peptides, 1,476 (1,465) protein (groups).<br>HEK-293: 32,794 precursors, 29,947 peptides, 5,517 (3,448) protein (groups).<br>HeLa: 33,392 precursors, 28,674 peptides, 4,954 (3,120) protein (groups).<br>*S. cerevisiae*: 22,240 precursors, 19,101 peptides, 2,384 (2,331) protein (groups).
MixPredicted[a]	Generated from precursors in MixStandard. Predicted using the model trained with HeLa1.<br>*C. elegans*: 18,808 precursors, 17,483 peptides, 3,554 (2,652) protein (groups).<br>*E. coli*: 14,583 precursors, 12,433 peptides, 1,474 (1,463) protein (groups).<br>HEK-293: 29,633 precursors, 27,150 peptides, 5,594 (3,416) protein (groups).<br>HeLa: 30,462 precursors, 27,259 peptides, 5,020 (3,108) protein (groups).<br>*S. cerevisiae*: 22,925 precursors, 18,684 peptides, 2,379 (2,329) protein (groups).
MixProsit[b]	Generated from precursors in MixStandard. Predicted using Prosit (CE30).<br>*C. elegans*: 18,745 precursors, 17,420 peptides, 3,554 (2,652) protein (groups).<br>*E. coli*: 14,473 precursors, 12,326 peptides, 1,474 (1,463) protein (groups).<br>HEK-293: 29,241 precursors, 26,764 peptides, 5,599 (3,410) protein (groups).<br>HeLa: 30,207 precursors, 27,005 peptides, 5,029 (3,103) protein (groups).

	S. cerevisiae: 22,732 precursors, 18,469 peptides, 2,380 (2,329) protein (groups).
HeLaProt[c]	Generated from tryptic peptides of 6,180 proteins in HeLaDDA, with 6,721 proteins in SwissProt *S. cerevisiae* database as entrapment. Predicted using the model trained with HeLa1.<br>HeLa: 407,969 precursors, 207,061 peptides, 6,173 (6,158) protein (groups).<br>*S. cerevisiae*: 306,469 precursors, 155,776 peptides, 6,622 (6,547) protein (groups).
PanProt[c]	Generated from tryptic peptides of totally 10,666 proteins in Pan and HeLaDDA, with 10,762 proteins in SwissProt *S. cerevisiae* (6,721) and *C. elegans* (4,041) databases as entrapment. Predicted using the model trained with HeLa1.<br>Human: 707,327 precursors, 358,849 peptides, 10,639 (10,617) protein (groups).<br>*S. cerevisiae*: 306,469 precursors, 155,776 peptides, 6,622 (6,547) protein (groups).<br>*C. elegans*: 226,163 precursors, 114,727 peptides, 4,027 (4,022) protein (groups).
HumanProt[c]	Generated from tryptic peptides of 20,282 proteins in SwissProt *Homo sapiens* database, with 19,388 proteins in SwissProt *S. cerevisiae* (6,721), *C. elegans* (4,041), *E. coli* (4,480) and *Dictyostelium discoideum* (4,146) databases as entrapment. Predicted using the model trained with HeLa1.<br>Human: 1,155,656 precursors, 585,934 peptides, 20,163 (20,091) protein (groups).<br>*S. cerevisiae*: 306,469 precursors, 155,776 peptides, 6,622 (6,547) protein (groups).<br>*C. elegans*: 226,163 precursors, 114,727 peptides, 4,027 (4,022) protein (groups).<br>*E. coli*: 143,706 precursors, 72,826 peptides, 4,464 (4,398) protein (groups).<br>*D. discoideum*: 220,660 precursors, 111,924 peptides, 4,113 (4,100) protein (groups).
HeLaProt50[d]	Generated from tryptic peptides with detectability score ≥ 0.5 of proteins in HeLaProt. Predicted using the model trained with HeLa1.<br>HeLa: 319,631 precursors, 161,376 peptides, 6,151 (6,123) protein (groups).<br>*S. cerevisiae*: 256,143 precursors, 129,500 peptides, 6,459 (6,347) protein (groups).
PanProt50[d]	Generated from tryptic peptides with detectability score ≥ 0.5 of proteins in PanProt. Predicted using the model trained with HeLa1.<br>Human: 541,212 precursors, 273,050 peptides, 10,591 (10,554) protein (groups).<br>*S. cerevisiae*: 256,143 precursors, 129,500 peptides, 6,459 (6,347) protein (groups).<br>*C. elegans*: 181,005 precursors, 91,290 peptides, 3,999 (3,988) protein (groups).
HumanProt50[d]	Generated from tryptic peptides with detectability score ≥ 0.5 of proteins in HumanProt. Predicted using the model trained with HeLa1.<br>Human: 856,075 precursors, 431,624 peptides, 19,841 (19,725) protein (groups).<br>*S. cerevisiae*: 256,143 precursors, 129,500 peptides, 6,459 (6,347) protein (groups).<br>*C. elegans*: 181,005 precursors, 91,290 peptides, 3,999 (3,988) protein (groups).<br>*E. coli*: 145,430 precursors, 73,541 peptides, 4,385 (4,319) protein (groups).<br>*D. discoideum*: 241,156 precursors, 121,794 peptides, 4,066 (4,045) protein (groups).
MouseProt[c]	Generated from tryptic peptides of 17,006 proteins in SwissProt *Mus musculus* database, with. 19,388 proteins in SwissProt *S. cerevisiae*

	(6,721), *C. elegans* (4,041), *E. coli* (4,480) and *D. discoideum* (4,146) databases as entrapment. Predicted using the model trained with HeLa1.<br>Mouse: 924,333 precursors, 469,159 peptides, 16,950 (16,927) protein (groups).<br>*S. cerevisiae*: 306,469 precursors, 155,776 peptides, 6,622 (6,547) protein (groups).<br>*C. elegans*: 226,163 precursors, 114,727 peptides, 4,027 (4,022) protein (groups).<br>*E. coli*: 143,706 precursors, 72,826 peptides, 4,464 (4,398) protein (groups).<br>*D. discoideum*: 220,660 precursors, 111,924 peptides, 4,113 (4,100) protein (groups).
MouseProt50[d]	Generated from tryptic peptides with detectability score ≥ 0.5 of proteins in MouseProt. Predicted using the model trained with HeLa1.<br>Mouse: 749,328 precursors, 377,694 peptides, 16,805 (16,727) protein (groups).<br>*S. cerevisiae*: 256,143 precursors, 129,500 peptides, 6,459 (6,347) protein (groups).<br>*C. elegans*: 181,005 precursors, 91,290 peptides, 3,999 (3,988) protein (groups).<br>*E. coli*: 145,430 precursors, 73,541 peptides, 4,385 (4,319) protein (groups).<br>*D. discoideum*: 241,156 precursors, 121,794 peptides, 4,066 (4,045) protein (groups).
MouseProt60[d]	Generated from tryptic peptides with detectability score ≥ 0.6 of proteins in MouseProt. Predicted using the model trained with HeLa1.<br>Mouse: 638,747 precursors, 321,665 peptides, 16,737 (16,656) protein (groups).<br>*S. cerevisiae*: 222,157 precursors, 111,978 peptides, 6,424 (6,340) protein (groups).<br>*C. elegans*: 155,192 precursors, 78,190 peptides, 3,989 (3,979) protein (groups).<br>*E. coli*: 97,922 precursors, 49,361 peptides, 4,297 (4,235) protein (groups).<br>*D. discoideum*: 159,575 precursors, 80,434 peptides, 3,954 (3,944) protein (groups).
MouseProt75[d]	Generated from tryptic peptides with detectability score ≥ 0.75 of proteins in MouseProt. Predicted using the model trained with HeLa1.<br>Mouse: 415,838 precursors, 208,854 peptides, 16,436 (16,339) protein (groups).<br>*S. cerevisiae*: 151,112 precursors, 75,957 peptides, 6,252 (6,165) protein (groups).<br>*C. elegans*: 103,316 precursors, 51,912 peptides, 3,942 (3,923) protein (groups).<br>*E. coli*: 68,088 precursors, 34,204 peptides, 4,157 (4,095) protein (groups).<br>*D. discoideum*: 110,858 precursors, 55,740 peptides, 3,890 (3,878) protein (groups).
SerumDDA	Generated from search results of fractionated DDA of serum samples with high-abundant protein (HAP) depletion.<br>10,591 precursors, 7,484 peptides, 956 (877) protein (groups).
PlasmaPredicted[a]	Generated from 27,248 peptides of 2,615 proteins identified from human plasma/serum data from previous projects in our labs. Predicted using the model for the Q Exactive HF in our lab trained with data collected from previous projects of different organisms. The model and training data are available at ProteomeXchange/iProX with the dataset identifier PXD014108/IPX0001628000.<br>52,913 precursors, 27,142 peptides, 2,656 (2,543) protein (groups).

[a] Libraries predicted with DeepDIA only contain precursors with 2+/3+ charge states of peptide with length from 7 to 50, and without any modification except carbamidomethyl cystine.

[b] Libraries predicted with Prosit only contain precursors with 2+/3+ charge states of peptides with

length from 7 to 30, and without any modification except carbamidomethyl cystine. Spectronaut applies filters to the imported spectral libraries, leading to different numbers of precursors/peptides in the libraries of different CEs.

[c] Large libraries without detectability filtering only contain tryptic peptides with length from 7 to 50 and mass ≤ 6000, digested with Trypsin and Trypsin/P without missed cleavages. The libraries only contain precursors with 2+/3+ charge states, and without any modification except carbamidomethyl cystine.

[d] Large libraries with detectability filtering only contain tryptic peptides with length from 7 to 50 and mass ≤ 6000, digested with Trypsin/P with missed cleavages ≤ 2. The libraries only contain precursors with 2+/3+ charge states, and without any modification except carbamidomethyl cystine. If all the peptides of a protein group are ruled out by detectability filtering, the protein group will be absent in the spectral library, leading to different numbers of protein (groups) in the libraries with different detectability thresholds.

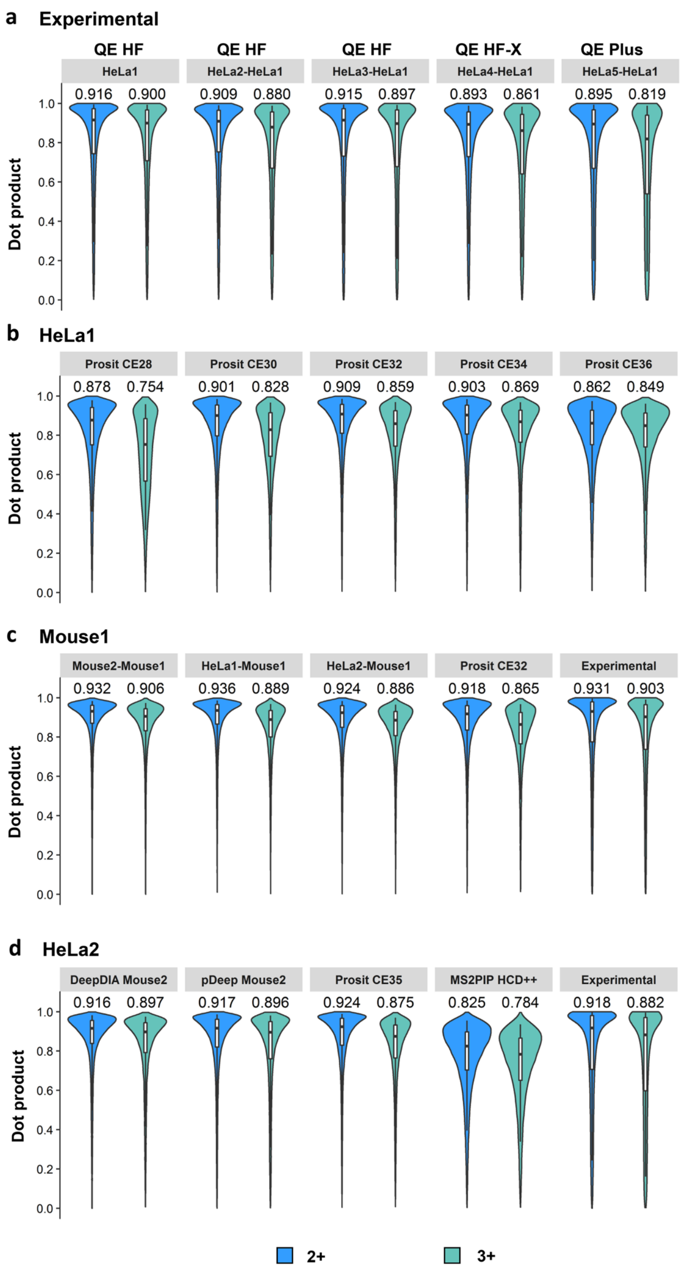

Supplementary Fig. 1. (**a**) The distributions of the dot products (DP) computed between experimental b/y/neutral loss peak intensities of the same precursors across several HeLa datasets from different labs. HeLa2 and HeLa3 were acquired on Q Exactive HF (QE HF) mass spectrometers in different labs from HeLa1. HeLa4 was acquired on Q Exactive HF-X (QE HF-X), and HeLa5 was acquired on Q Exactive Plus (QE Plus). (**b**) The distributions of the DP computed between experimental b/y/neutral loss peak intensities from the HeLa1 dataset and predicted intensities by Prosit with different normalized collision energy (CE) parameters. (**c**) The distributions of dot products computed between predicted and experimental b/y/neutral loss peak intensities from Mouse1 data. The CE parameter was optimized for Prosit on the data. (**d**) Performance comparison of DeepDIA and existing tools for peptide MS/MS spectrum prediction. The CE parameter was optimized for Prosit on the data. The medians are indicated. The boxes and whiskers show the quantiles and 95% percentiles, respectively. 2+: doubly-charged precursors; 3+: triply-charged precursors. Source data are provided as a Source Data file.

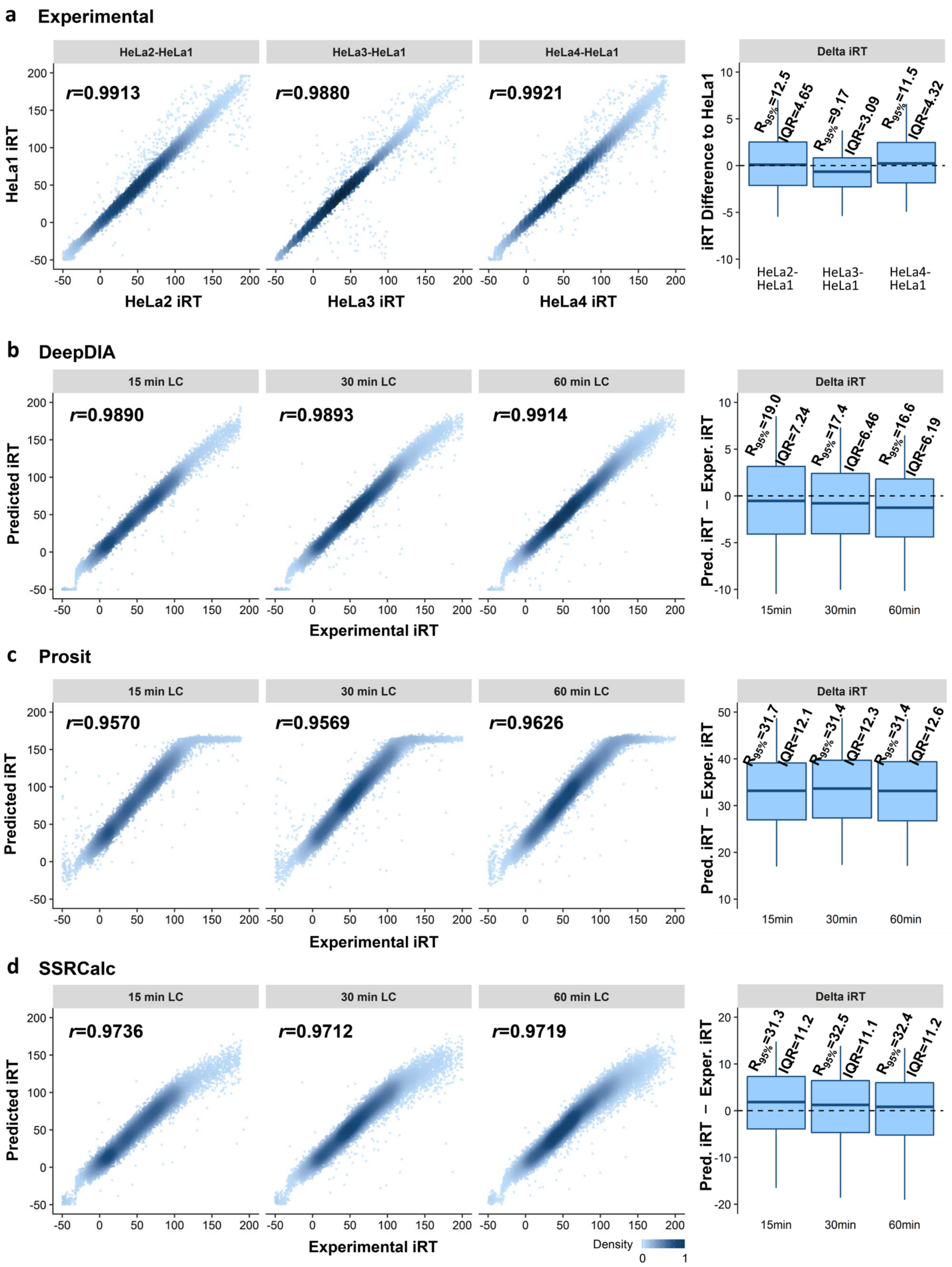

Supplementary Fig. 2. (**a**) Pearson correlation coefficients (*r*) and the differences (Delta iRT) computed between experimental normalized retention time (iRT) of the same peptides across several HeLa datasets from different labs. (**b-d**) Pearson correlation coefficients (*r*) and the differences (Delta iRT) computed between experimental iRT and predicted iRT using DeepDIA, Prosit and SSRCalc. Experimental data were from HeLa cells (HeLa2) acquired on a Q Exactive HF mass spectrometer with different liquid chromatography (LC) gradients. The model that DeepDIA used was trained with Mouse2 data. Color gradation indicates relative density of data points. The boxes show interquartile ranges (IQR), and the whiskers show 95% percentiles; no outliers are shown. Source data are provided as a Source Data file.

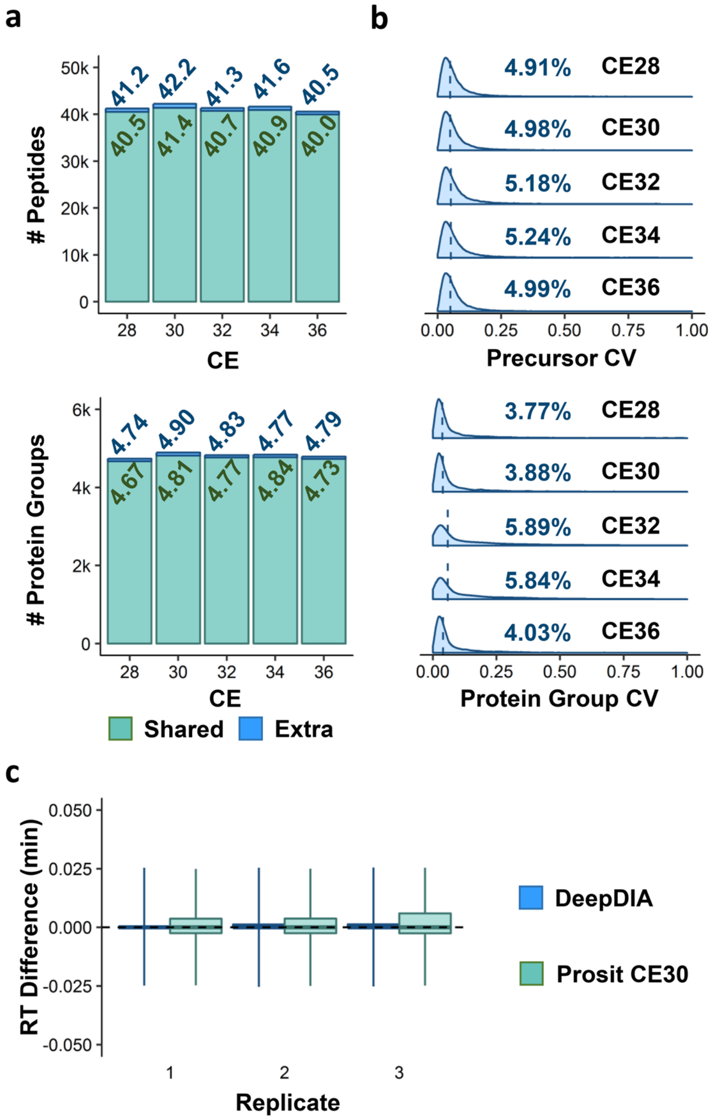

Supplementary Fig. 3. (**a**) The numbers of peptides and protein groups detected using the HeLaProsit libraries with different normalized collision energy (CE) parameters. Overlapping identifications with the DDA library are referred as "shared". (**b**) The distributions of coefficient of variation (CV) of peptide precursor and protein group quantification results detected in three technical replicates using the HeLaProsit libraries with different CE parameters. The medians are indicated. (**c**) Differences between the apex RTs of precursors detected using the predicted libraries and those using the HeLaDDA library. The boxes show interquartile ranges (IQR), and the whiskers show 95% percentiles; no outliers are shown. Details of the spectral libraries are described in **Supplementary Table 2**. Source data are provided as a Source Data file.

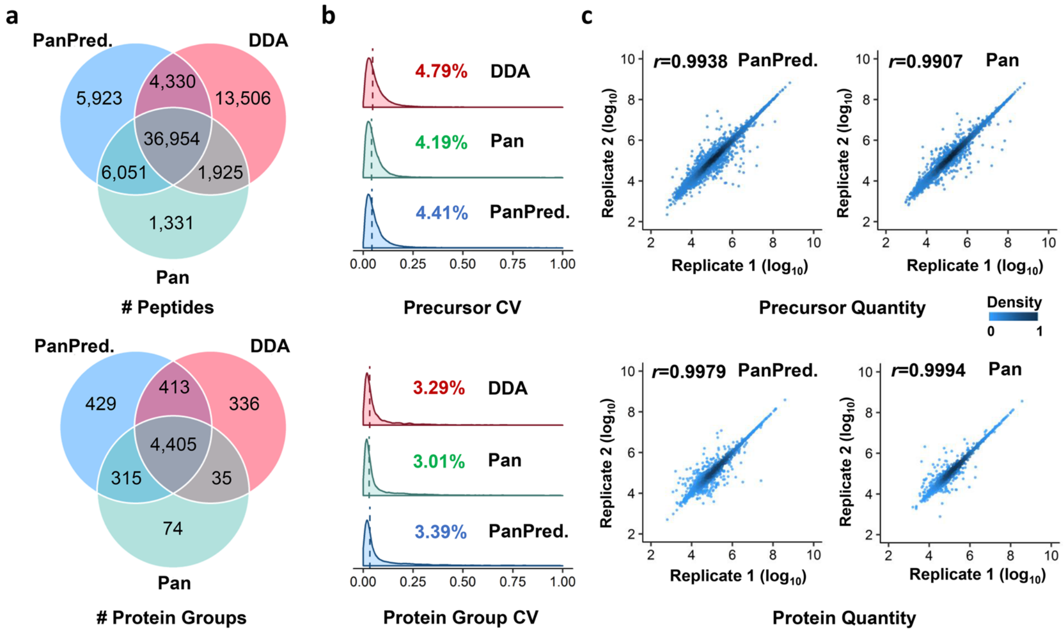

Supplementary Fig. 4. Performance comparison of the PanPredicted (PanPred.), the Pan, and the HeLaDDA (DDA) libraries on a dataset of HeLa cells (HeLa1). (**a**) The numbers of peptides and protein groups detected. (**b**) The distributions of coefficient of variation (CV) of peptide precursor and protein group quantification results detected in three technical replicates using the three libraries. The medians are indicated. (**c**) Correlation between replicates of precursor and protein group quantification results. *r* stands for Pearson correlation coefficients. Color gradation indicates relative density of data points. Details of the HeLa1 dataset are described in **Supplementary Table 1**. Details of the spectral libraries are described in **Supplementary Table 2**. Source data are provided as a Source Data file.

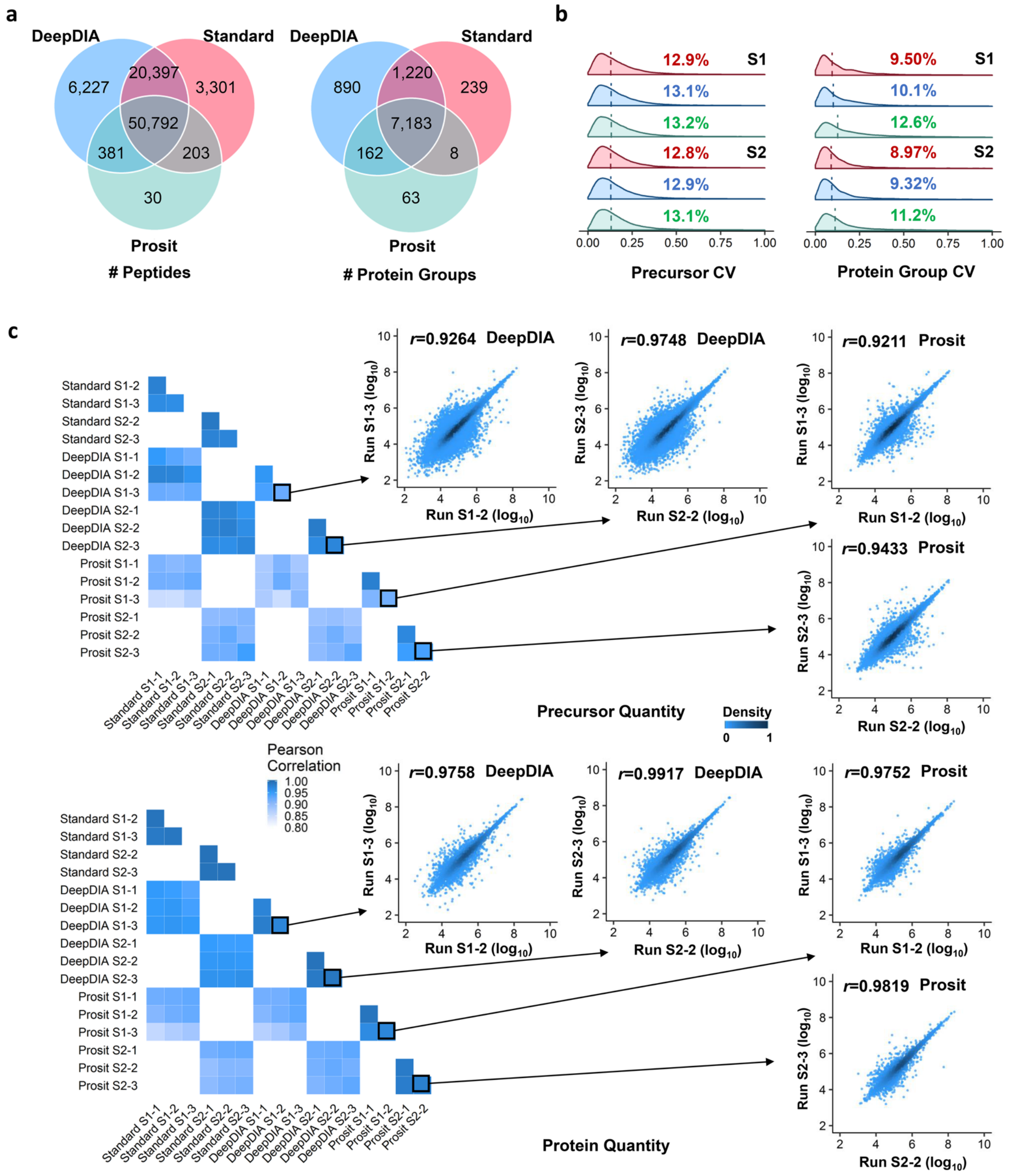

Supplementary Fig. 5. Performance comparison of the *in silico* libraries predicted by DeepDIA and Prosit, and the DDA library on a dataset of mix proteome samples (Mix). (**a**) The numbers of peptides and protein groups detected using the standard DDA library (Standard: MixStandard), the predicted library by DeepDIA (DeepDIA: MixPredicted), and the predicted library by Prosit (Prosit: MixProsit). Prosit library was built with the optimal normalized collision energy (CE = 30). Among the detected peptides, 1,284 (1,152 using MixStandard and 627 using MixPredicted) with length > 30 were absent in the Prosit library. (**b**) The distributions of coefficient of variation (CV) of peptide precursor and protein group quantification results in three technical replicates of each sample using the three libraries. The medians are indicated. (**c**) Correlation between replicates of precursor and protein group quantification results. *r* stands for Pearson correlation coefficients. Color gradation in correlation plots indicates relative density of data points. Details of the Mix dataset are described in **Supplementary Table 1**. Details of the spectral libraries are described in **Supplementary Table 2**. Source data are provided as a Source Data file.

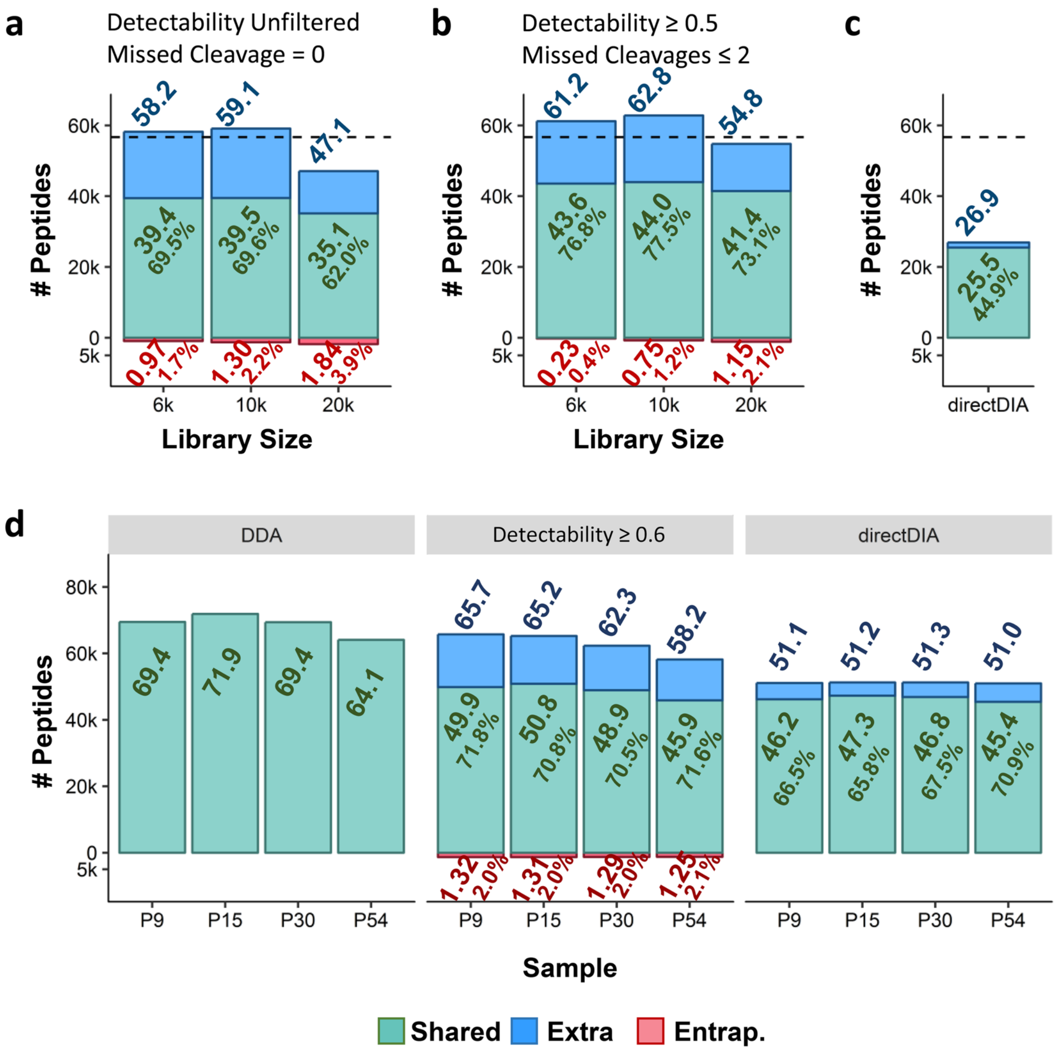

Supplementary Fig. 6. DIA analysis results using large *in silico* spectral libraries. (**a**) The numbers of peptides detected from HeLa1 using HeLaProt (6k proteins), PanProt (10k proteins), and HumanProt (20k proteins). The libraries include all *in-silico* tryptic digested peptides without missed cleavage. (**b**) The numbers of peptides detected from HeLa1 using HeLaProt50 (6k proteins), PanProt50 (10k proteins), and HumanProt50 (20k proteins). The libraries include *in-silico* tryptic digested peptides with ≤ 2 missed cleavages and a detectability score ≥ 0.5. (**c**) The numbers of peptides detected from HeLa1 using directDIA. (**d**) The numbers of peptides detected from Mouse1 using the DDA-based library, MouseProt60 (an *in silico* library generated from SwissProt *Mus musculus* database with a detectability score threshold of 0.6 and ≤ 2 missed cleavages), and directDIA. The dashed lines indicate the numbers of peptides detected using the DDA-based libraries. Overlapping identifications with the DDA-based libraries are referred as "shared". Peptide numbers, sensitivities and entrapment (Entrap.) percentages are indicated. Details of the HeLa1 and Mouse1 datasets are described in **Supplementary Table 1**. Details of the spectral libraries are described in **Supplementary Table 2**.

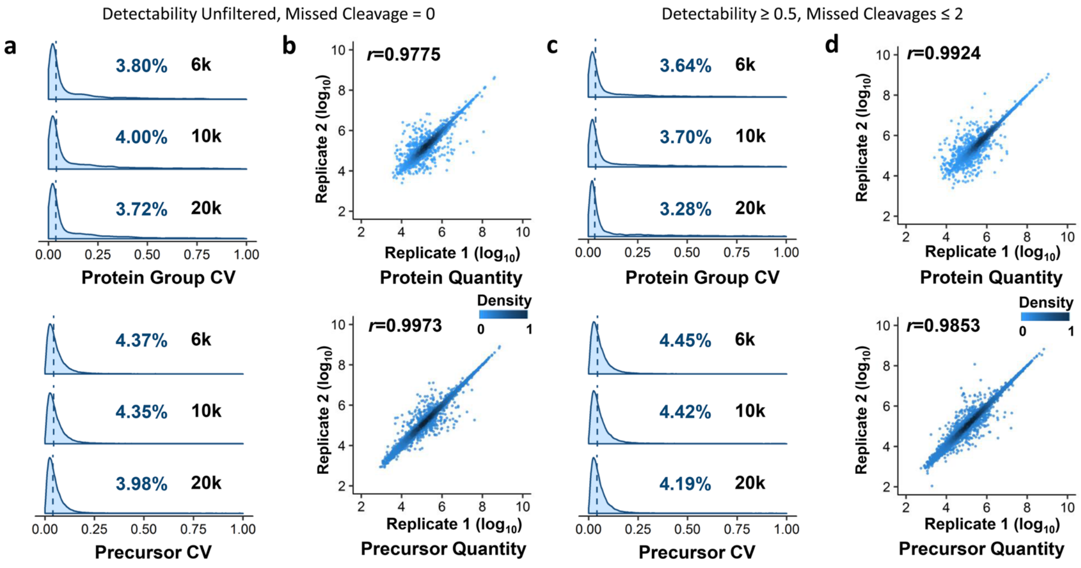

Supplementary Fig. 7. DIA analysis results of a dataset of HeLa cells (HeLa1) using large predicted libraries. (**a**) The distributions of coefficient of variation (CV) of protein group and precursor quantification results in three technical replicates using the HeLaProt (6k), PanProt (10k), and HumanProt (20k) library. (**b**) Correlation between replicates of protein group and precursor quantification results using the HumanProt (20k) library. (**c**) The distributions of CV of protein group and precursor quantification results in three technical replicates using the HeLaProt50 (6k), PanProt50 (10k), and HumanProt50 (20k) library. (**d**) Correlation between replicates of protein group and precursor quantification results using the HumanProt50 (20k) library. *r* stands for Pearson correlation coefficients. Color gradation indicates relative density of data points. Details of the HeLa1 dataset are described in **Supplementary Table 1**. Details of the spectral libraries are described in **Supplementary Table 2**. Source data are provided as a Source Data file.

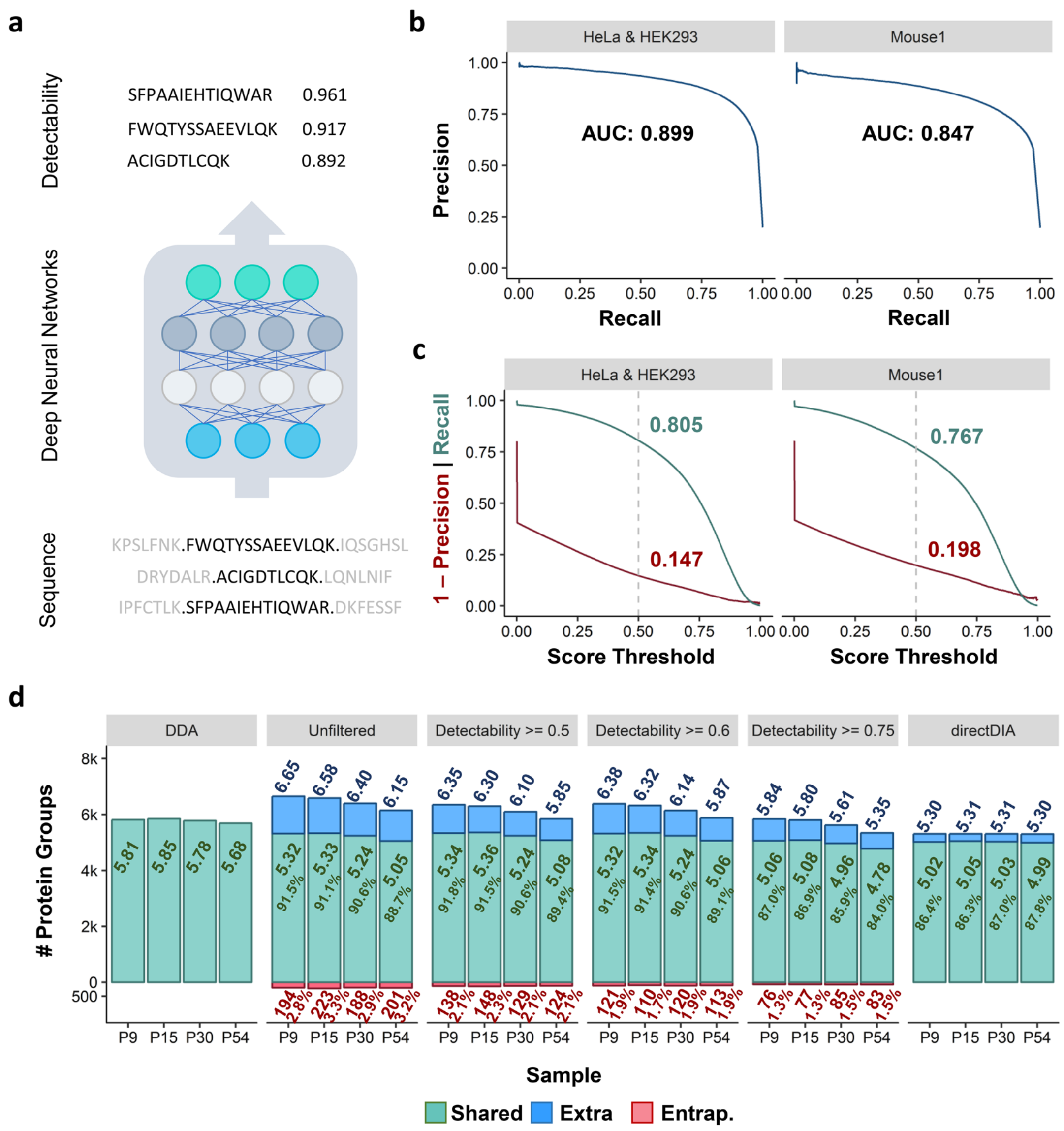

Supplementary Fig. 8. Prediction of peptide detectability by mass spectrometry. (**a**) A graphical illustration of the deep neural networks for peptide detectability prediction. (**b**) Precision-recall (PR) curves of peptide detectability prediction on datasets of HeLa and HEK-293 cells (HeLa&HEK, **Supplementary Table 1**) and mouse tissue (Mouse1, **Supplementary Table 1**). (**c**) Precision and recall of detectability prediction against detectability scores. (**d**) The numbers of protein groups detected using the DDA-based library, *in silico* libraries generated from SwissProt *Mus musculus* database without detectability filtering (MouseProt) or with different detectability score thresholds (MouseProt50, MouseProt60, MouseProt75), and directDIA. Tryptic specific digested peptides without missed cleavage were considered for the unfiltered library, and those with ≤ 2 missed cleavages were considered for the libraries with detectability filtering. Overlapping identifications with the DDA-based libraries are referred as “shared”. Protein group numbers, sensitivities and entrapment (Entrap.) percentages are indicated. Details of the spectral libraries are described in **Supplementary Table 2**. Source data are provided as a Source Data file.

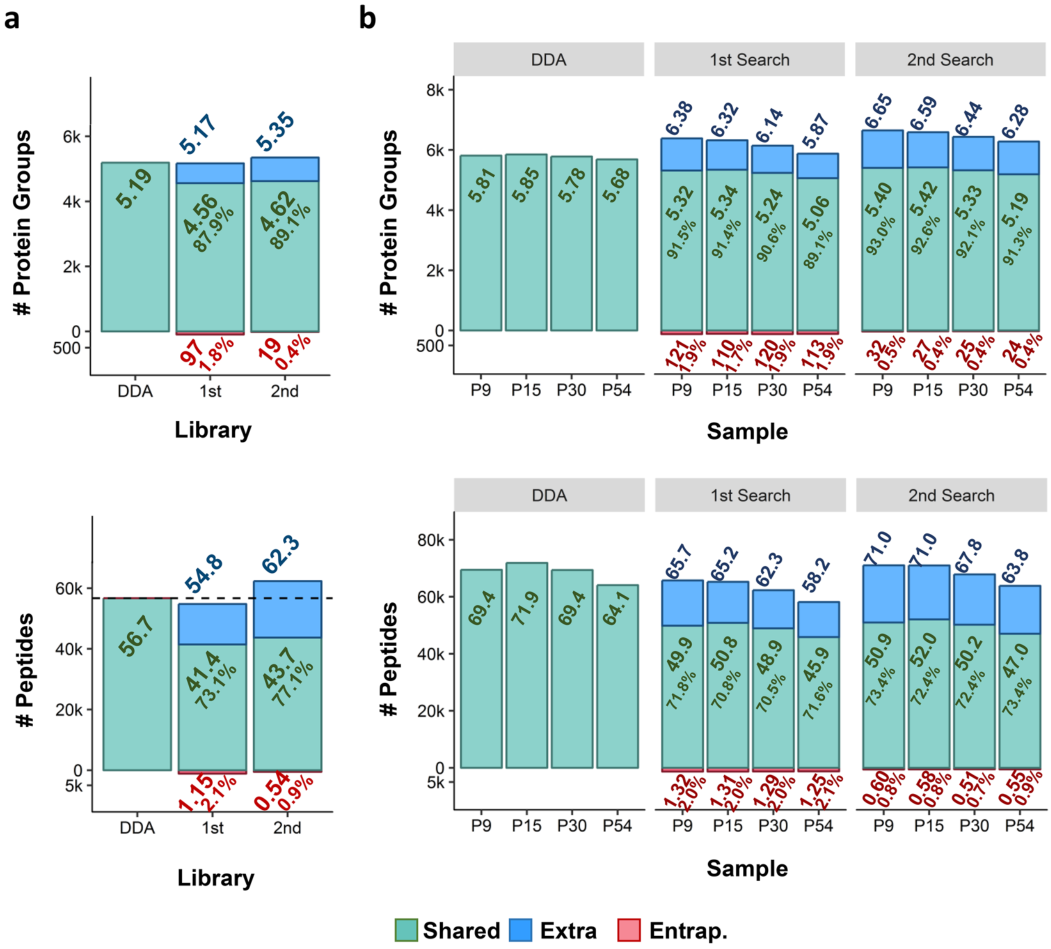

Supplementary Fig. 9. DIA analysis results using a two-step approach. (**a**) The numbers of protein groups and peptides detected from the HeLa1 (**Supplementary Table 1**) dataset. (**b**) The numbers of protein groups and peptides detected from the Mouse1 (**Supplementary Table 1**) dataset. The library used for the second search on HeLa1 was generated from the first search result using HumanProt50 *in silico* spectral library. Protein inference was re-performed on the *in silico* digested peptides of the proteins detected in the first search with ≤ 2 missed cleavages and with detectability score ≥ 0.5, and consequently the library contained 5,831 proteins (5,792 protein groups) from *H. sapiens*, while 6,459 proteins (6,347 protein groups) from *S. cerevisiae* and 4,385 proteins (4,319 protein groups) from *E. coli* were add as entrapment. The library used for the second search on Mouse1 was generated from the first search results using MouseProt60 *in silico* spectral library (combined from four groups of samples), containing 7,424 proteins (7,380 protein groups) from *M. musculus*, while 6,424 proteins (6,340 protein groups) from *S. cerevisiae* and 4,297 proteins (4,235 protein groups) from *E. coli* were add as entrapment. Overlapping identifications with the DDA-based libraries are referred as “shared”. Protein group/peptide numbers, sensitivities and entrapment (Entrap.) percentages are indicated. Details of the spectral libraries are described in **Supplementary Table 2**.

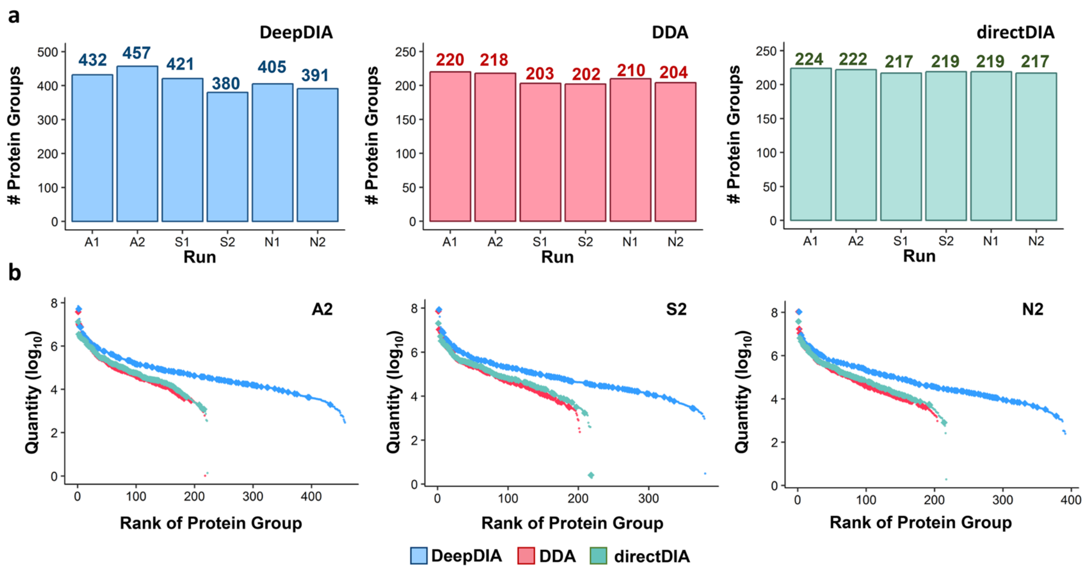

Supplementary Fig. 10. DIA analysis results of a dataset of human serum (Serum). (**a**) The numbers of protein groups detected using the predicted library (PlasmaPredicted, DeepDIA), the DDA-based library (SerumDDA, DDA), and directDIA, respectively. (**b**) Plots of protein group quantity (log_{10}) against rank of protein group by quantification results using the predicted library, the DDA-based library and directDIA in A2, S2 and N2, respectively. A small cycle represents a protein group and a large diamond represents a PQ500 protein group. A, S and N represent the three serum samples, respectively. A2, S2 and N2 were mixed with PQ500 Reference Peptides Kit. Details of the Serum dataset are described in **Supplementary Table 1**. Details of the spectral libraries are described in **Supplementary Table 2**. Source data are provided as a Source Data file.

Supplementary Note 1. Evaluation of reproducibility of peptide MS/MS spectra and RT across instruments and labs

We collected DDA data of HeLa cells from five Orbitrap mass spectrometers in different labs. HeLa1[1] (ProteomeXchange identifier PXD005573, **Supplementary Table 1**) were acquired on a Q Exactive HF mass spectrometer. HeLa2[2] (ProteomeXchange identifier PXD006932, **Supplementary Table 1**) and HeLa3[3] (ProteomeXchange identifier PXD004452) were acquired on Q Exactive HF mass spectrometers in different labs from HeLa1. HeLa4[2] (ProteomeXchange identifier PXD006932) was acquired on a Q Exactive HF-X mass spectrometer, and HeLa5[4] (ProteomeXchange identifier PXD009875) was acquired on Q Exactive Plus. All the DDA data were analyzed with SpectroMine as described in the Methods section. We collected MS/MS spectra of 69,577 peptides (57,198 doubly-charged precursors and 27,468 triply-charged precursors), 88,462 peptides (67,053 doubly-charged precursors and 37,480 triply-charged precursors), 142,360 peptides (110,756 doubly-charged precursors and 76,915 triply-charged precursors), 94,884 peptides (74,475 doubly-charged precursors and 42,941 triply-charged precursors), and 51,571 peptides (41,444 doubly-charged precursors and 18,898 triply-charged precursors) from HeLa1, HeLa2, HeLa3, HeLa4 and HeLa5, respectively. HeLa2, HeLa3, HeLa4 and HeLa5 shared 36,668 peptides (31,329 doubly-charged precursors and 12,284 triply-charged precursors), 40,936 peptides (34,676 doubly-charged precursors and 14,995 triply-charged precursors), 39,527 peptides (33,584 doubly-charged precursors and 13,493 triply-charged precursors), and 35,438 peptides (29,487 doubly-charged

precursors and 11,555 triply-charged precursors) with HeLa1, respectively. Dot products[5] (DP) were computed between peak intensities of b/y/neutral loss product ions of the same precursors in HeLa1 and the other datasets (**Supplementary Fig. 1a**). Similarities of peptide MS/MS spectra acquired on Q Exactive HF across different labs (HeLa2-HeLa1 and HeLa3-HeLa1) were higher than those across different types of Orbitrap mass spectrometers (HeLa4-HeLa1 and HeLa5-HeLa1), but were lower than those acquired on the same instrument (HeLa1). Pearson correlation coefficients (*r*) of iRT and iRT differences were also computed between HeLa1 and the other datasets (**Supplementary Fig. 2a**).

Supplementary Note 2. Performance comparison of DeepDIA and existing tools for peptide MS/MS spectrum prediction

The performance of peptide MS/MS prediction of DeepDIA was compared with Prosit[6] (access date 2019-08, https://www.proteomicsdb.org/prosit/) on higher energy collisional dissociation (HCD) spectra from the HeLa1 dataset. To optimize the collision energy (CE) parameter for Prosit, three RAW files (because file size is limited to 2GB by Prosit online service) with corresponding MaxQuant results (version 1.5.2.8; searched against SwissProt *H. sapiens* database; no variable modifications were specified; other parameters were default) were uploaded, and the optimal CE was 32 for all the RAW files. DPs between the experimental and predicted peak intensities are shown in **Supplementary Fig. 1b**. DP by Prosit with the best calibration CE was still lower than DeepDIA with models trained with HeLa1, HeLa2 (in a different lab) and Mouse1 (on the same instrument, **Supplementary Table 1**) (**Fig. 2c**). We also performed comparison on a dataset of mouse tissue[1] (Mouse1). DP by Prosit was lower than DeepDIA with models trained with Mouse2 (on a different instrument in the same lab, **Supplementary Table 1**), HeLa1 (on the same instrument) and HeLa2 (in a different lab).

We further compared DeepDIA with Prosit, MS^{2}PIP[7] (access date 2019-03, https://iomics.ugent.be/ms2pip/) and pDeep[8] (access date 2017-11, https://github.com/pFindStudio/pDeep) on HCD spectra from the HeLa2 dataset. For Prosit, CE value was optimized to 35. For MS^{2}PIP prediction, HCD (including b++ and y++ ions, version 20190107) was specified as the model. For DeepDIA and

pDeep, deep neural networks were trained with the Mouse2 dataset (different organism and in a different lab). The spectra predicted using DeepDIA showed median dot product scores close to pDeep, slight worse than Prosit for 2+ precursors, and better than Prosit for 3+ precursors. All the deep learning-base methods outperformed MS^2PIP.

Supplementary Note 3. Performance comparison of DeepDIA and existing tools for peptide iRT prediction

The performance of DeepDIA, Prosit and SSRCalc[9] (version Q, access date 2019-03, http://hs2.proteome.ca/SSRCalc/SSRCalcQ.html) for RT prediction was evaluated on DDA data from the HeLa2 dataset, with LC gradient lengths ranging from 15 min to 1 h. For DeepDIA, deep neural networks were trained with Mouse2 dataset (cross-organism and cross-lab). For Prosit, CE was set to 35 and precursor charge was set to 2+. For SSRCalc, separation system was set as 100Å C18 column and 0.1% Formic Acid. iRT was calculated from hydrophobicity indexes (HI) using the correlation function $iRT = -44.29 + 7.25 \times HI$, which was estimated using the standard iRT peptides (iRT Kit, Biognosys AG, Schlieren, Switzerland). Using DeepDIA, Pearson correlation coefficients (r) of predicted and experimental iRT were higher than Prosit and SSRCalc, while the interquartile ranges (IQR) of the differences between predicted and experimental iRT were smaller than Prosit and SSRCalc. Currently, Prosit website does not offer iRT refinement service, and thus prediction was performed with the existing Prosit model trained on the ProteomeTools data. We believe that prediction accuracy would be improved once refinement was performed by retraining the model, as reported in their publications. Indeed, training instrument-specific models is exactly what we are suggesting.

Supplementary Note 4. Performance comparison of DeepDIA and Prosit on the datasets of HeLa cells and mixed proteome samples

The performance of DeepDIA was compared with Prosit for DIA analysis on a dataset of HeLa cells (HeLa1). *In silico* spectral libraries were generated using DeepDIA (HeLaPredicted, see **Supplementary Table 2** for details) and Prosit (HeLaProsit, **Supplementary Table 2**), respectively. For MS/MS prediction by Prosit, different CEs were used (**Supplementary Fig. 3a** and **3b**). Numbers of detected peptides and protein groups were maximized when CE = 30. 29% more peptides (excluding those with length > 30, which is not supported by Prosit) and 6% more protein groups were detected by HeLaPredicted than HeLaProsit (**Fig. 3a**). The median coefficients of variation (CVs) of peptide precursor and protein group quantification results detected in three technical replicates using the HeLaPredicted library were smaller than those using the HeLaProsit library (**Fig. 3b**).

DeepDIA and Prosit were also compared on a dataset of mixed proteome samples[1] containing peptides from *Homo sapiens*, *Caenorhabditis elegans*, *Saccharomyces cerevisiae* and *Escherichia coli* with different abundance (see **Supplementary Table 1** for details). *In silico* spectral libraries were generated using DeepDIA (MixPredicted, see **Supplementary Table 2** for details) and Prosit (MixProsit, **Supplementary Table 2**), respectively. For Prosit, CE was optimized to 30. MixPredicted led to detection of 51% more peptides (excluding those with length > 30) and 27% more protein groups than MixProsit, decreased the CVs, and improved r of precursor and protein group quantification results among replicates (**Supplementary Fig. 5**). Percent changes of

detected precursors and protein groups of each organism between the two samples were computed based on the mean quantities in three replicates of each sample. The percent changes estimated using the MixPredicted library were more accurate than those using the MixProsit library, at both precursor and protein group level (**Fig. 3d**).

Supplementary Note 5. Prediction of peptide detectability by mass spectrometry using deep learning

Peptides of proteins identified with sequence coverage ≥ 25% were collected from a dataset of HeLa and HEK-293 cells[1] (HeLa&HEK, **Supplementary Table 1**). For DDA data of peptides without fractionation, peptide quantities were normalized to the peptide with maximum quantity in each protein. For most of the peptides, relative quantities (RQ) were from 10^{-5} to 1, and thus detectability scores (D) were calculated by

$$D = \max[(5 + \log_{10} \mathrm{RQ}) / 10 + 0.5, 0.5] \quad (1)$$

For the peptides in the HeLa&HEK dataset without fractionation, the calculated D was from 0.654 to 1. For DDA data of peptides with high pH reversed phase fractionation, RQ of peptides are not comparable across different fractions. Based on the assumption that peptides observed in DDA without fractionation are more likely to be detected than those only observed in DDA with fractionation, detectability scores of peptides only observed with fractionation were set to the average of 0.5 and the minimum of detectability scores of those detected without fractionation, *e.g.* (0.5 + 0.654) / 2 = 0.577 for the HeLa&HEK dataset. Thus, D of all the detected peptides were ≥ 0.5. Tryptic specific *in silico* digested peptides (Trypsin/P, missed cleavages ≤ 2, length from 7 to 50 amino acids, mass ≤ 6,000 Da) that were not experimentally observed were collected as negative datasets, and their detectability scores were set to 0. As a result, 2,691 proteins (61,240 positive and 247,040 negative peptides) were obtained from the HeLa&HEK dataset. The same rule was applied on a dataset of

mouse tissue (Mouse1), wherein 53,676 positive and 219,820 negative peptides of 2,363 proteins were obtained.

Due to the unbalance of positive and negative data, a hard negative mining[10] approach was used to train the model. First, a subset of the negative data with equivalent size to the positive data was randomly selected, and the model was trained with all the positive data and the negative subset. Next, all the negative data were reranked by predicted detectability scores (between 0 and 1) in descending order, and a subset of the negative data with the top detectability scores and equivalent size to the positive data was selected. The model was retrained with all the positive data and the new negative subset. This step was repeated until the accuracy of prediction was no longer improved.

Recalls, *i.e.* true positive / (true positive + false negative), and precision, *i.e.* true positive / (true positive + false positive) of prediction are shown in **Supplementary Fig. 8b** and **8c**. Using a model trained with the HeLa&HEK data, area under the precision-recall curve (AUPRC) of prediction was ~0.90 for HeLa&HEK, and ~0.85 for Mouse1 (cross-species). When a detectability score threshold of 0.5 was used to discriminate whether a peptide could be detected by mass spectrometry, recall was 0.80 and 0.76 for HeLa&HEK and Mouse1, while error rate (1 − precision) was 0.15 and 0.20 for HeLa&HEK and Mouse1, respectively. Increasing the threshold will increase the precision, but sacrifice the recall. In the context of spectral library building for DIA analysis, recall is more important than precision. Even though the error rates are not low, most negative peptides can be ruled out, and thus the query

space of DIA analysis is significantly reduced. During the DIA analysis by Spectronaut, by setting a Q-value the error rate can be further controlled.

Supplementary Note 6. Reducing the query space for DIA analysis

Generating spectral libraries containing only peptides likely to be detected can facilitate improvement of sensitivities and control of error rates for DIA analysis. *In silico* spectral libraries can be built from peptide lists in community libraries such as Pan-Human[11], public repositories like the global proteome machine database (GPMDB)[12], or with prior knowledge from previous studies, *e.g.* plasma in this study. However, in many cases, there is a lack of peptide-level prior knowledge, and spectral libraries can only be built from protein sequences. By predicting peptide detectability by mass spectrometry, target peptides can be selected from proteins with an appropriate detectability score threshold as discussed above.

Strategies for large-scale database searching such as iterative searching[13], two-step methods[14], and sectioning approaches[15] are worth a shot. Attempts were taken to adapt a two-step approach for DIA analysis using spectral libraries generated from proteome-scale databases, *e.g.* SwissProt. On the HeLa1 dataset, we downsized the spectral library generated from SwissProt *H. sapiens* (HumanProt50) to the proteins detected in the first search. Protein inference was re-performed on the *in silico* digested peptides of the proteins detected in the first search with ≤ 2 missed cleavages and with detectability score ≥ 0.5, and consequently the library contained 5,831 proteins (5,792 protein groups) from *H. sapiens*. 6,459 proteins (6,347 protein groups) from *S. cerevisiae* and 4,385 proteins (4,319 protein groups) from *E. coli* were added as entrapment. A second search was performed. The percentage of entrapment hits decreased from 1.8% to 0.4%, while the sensitivity increased to 89% on protein group

level. The number of identified protein groups and peptides also increased after the second search (**Supplementary Fig. 8**). Similar results were obtained on the Mouse1 dataset. During the second search, the library size was smaller and the library was more specific to the sample, which could lead to better performance in peptide and protein identification.

Supplementary Note 7. Running time for model training, library generation and DIA analysis

Expected running time depends on the size of data and the performance of the computer. In this study, model training and library generation was performed on a workstation with Intel Xeon E5-2690 v3 CPU, 16 GB RAM, and Microsoft Windows Server 2016 Version 1607 (OS Build 14393.2430) operating system. Training the model for MS/MS prediction with HeLa1 took ~5 h for 2+ precursors and ~4 h for 3+ precursors, respectively. Training the model for iRT prediction with HeLa1 took ~6 h. Training the model for peptide detectability prediction with HeLa&HEK took ~8 h. Generating the PanPredicted library took < 1 h. Generating the HumanProt library took 10 h. GPU cards with Compute Unified Device Architecture (CUDA) would highly accelerate the training and prediction process.

DIA data analysis was performed on a workstation with Intel Core i9-7960X CPU, 128 GB RAM, and Microsoft Windows 10 Version 1809 (OS Build 17763.503) 64-bit operating system. Analysis of the HeLa1 data using the PanPredicted and HumanProt library took < 1 h and ~3 h, respectively. On the HeLa1 data, directDIA analysis against SwissProt *H. sapiens* database took ~2 h with the same computer.

Supplementary References

1. Bruderer, R. et al. Optimization of experimental parameters in data-independent mass spectrometry significantly increases depth and reproducibility of results. *Mol. Cell. Proteomics* **16**, 2296-2309 (2017).
2. Kelstrup, C. D. et al. Performance evaluation of the Q Exactive HF-X for shotgun proteomics. *J. Proteome Res.* **17**, 727-738 (2018).
3. Bekker-Jensen, D. B. et al. An optimized shotgun strategy for the rapid generation of comprehensive human proteomes. *Cell Syst.* **4**, 587-599 (2017).
4. Zhang, Y., Wen, Z., Washburn, M. P. & Florens, L. Evaluating chromatographic approaches for the quantitative analysis of a human proteome on orbitrap-based mass spectrometry systems. *J. Proteome Res.* **18**, 1857-1869 (2019).
5. Wan, K. X., Vidavsky, I. & Gross, M. L. Comparing similar spectra: From similarity index to spectral contrast angle. *J. Am. Soc. Mass Spectrom.* **13**, 85-88 (2002).
6. Gessulat, S. et al. Prosit: Proteome-wide prediction of peptide tandem mass spectra by deep learning. *Nat. Methods* **16**, 509-518 (2019).
7. Degroeve, S., Maddelein, D. & Martens, L. MS2PIP prediction server: Compute and visualize MS2 peak intensity predictions for CID and HCD fragmentation. *Nucleic Acids Res.* **43**, W326-W330 (2015).
8. Zhou, X.-X. et al. pDeep: Predicting MS/MS spectra of peptides with deep learning. *Anal. Chem.* **89**, 12690-12697 (2017).
9. Krokhin, O. V. et al. An improved model for prediction of retention times of tryptic peptides in ion pair reversed-phase HPLC. *Mol. Cell. Proteomics* **3**, 908-919 (2004).
10. Felzenszwalb, P. F., Girshick, R. B., McAllester, D. & Ramanan, D. Object detection with discriminatively trained part-based models. *IEEE T. Pattern Anal.* **32**, 1627-1645 (2010).
11. Rosenberger, G. et al. A repository of assays to quantify 10,000 human proteins by SWATH-MS. *Sci. Data* **1**, 140031 (2014).
12. Shanmugam, A. K. & Nesvizhskii, A. I. Effective leveraging of targeted search spaces for improving peptide identification in tandem mass spectrometry based proteomics. *J. Proteome Res.* **14**, 5169-5178 (2015).
13. Rooijers, K. et al. An iterative workflow for mining the human intestinal metaproteome. *BMC Genomics* **12**, 6 (2011).
14. Jagtap, P. et al. A two-step database search method improves sensitivity in peptide sequence matches for metaproteomics and proteogenomics studies. *Proteomics* **13**, 1352-1357 (2013).
15. Muth, T. et al. Navigating through metaproteomics data: A logbook of database searching. *Proteomics* **15**, 3439-3453 (2015).
