# Supplementary material for: In silico spectral libraries by deep learning facilitate data-independent acquisition proteomics
Source: Nat Commun. 2020 Jan 9;11:146. doi: 10.1038/s41467-019-13866-z (PMC6952453; doi:10.1038/s41467-019-13866-z)
Supplement: Supplementary file 2 — Description of Additional Supplementary Files [file 41467_2019_13866_MOESM2_ESM.docx]

Description of Additional Supplementary Files

File Name: Supplementary Data 1

Description: DIA analysis results of a dataset of HeLa cells (HeLa1) using the DDA-based library, as well as libraries predicted by DeepDIA and Prosit.

File Name: Supplementary Data 2.

Description: DIA analysis results of a dataset of HeLa cells (HeLa1) using the Pan-Human library and the PanPredicted library.

File Name: Supplementary Data 3.

Description: DIA analysis results of a dataset of mixed proteome samples (Mix) using the DDA-based library, as well as libraries predicted by DeepDIA and Prosit.

File Name: Supplementary Data 4.

Description: DIA analysis results of a dataset of HeLa cells (HeLa1) using large libraries predicted by DeepDIA.

File Name: Supplementary Data 5.

Description: DIA analysis results of a dataset of mouse tissue (Mouse1) using the DDA-based library, as well as large libraries predicted by DeepDIA.

File Name: Supplementary Data 6.

Description: DIA analysis results of a dataset of human serum (Serum) using the DDA-based library and the PlasmaPredicted library predicted by DeepDIA.
