# Supplementary material for: In silico spectral libraries by deep learning facilitate data-independent acquisition proteomics
Source: Nat Commun. 2020 Jan 9;11:146. doi: 10.1038/s41467-019-13866-z (PMC6952453; doi:10.1038/s41467-019-13866-z)
Supplement: Supplementary file 9 — Reporting Summary [file 41467_2019_13866_MOESM9_ESM.pdf]

nature research

Corresponding author(s): Liang Qiao

Last updated by author(s): Nov 29, 2019

Reporting Summary

Nature Research wishes to improve the reproducibility of the work that we publish. This form provides structure for consistency and transparency in reporting. For further information on Nature Research policies, see Authors & Referees and the Editorial Policy Checklist .

Statistics

For all statistical analyses, confirm that the following items are present in the figure legend, table legend, main text, or Methods section.

n/a	Confirmed	
☐	☒	The exact sample size (*n*) for each experimental group/condition, given as a discrete number and unit of measurement
☐	☒	A statement on whether measurements were taken from distinct samples or whether the same sample was measured repeatedly
☒	☐	The statistical test(s) used AND whether they are one- or two-sided<br>*Only common tests should be described solely by name; describe more complex techniques in the Methods section.*
☒	☐	A description of all covariates tested
☒	☐	A description of any assumptions or corrections, such as tests of normality and adjustment for multiple comparisons
☐	☒	A full description of the statistical parameters including central tendency (e.g. means) or other basic estimates (e.g. regression coefficient) AND variation (e.g. standard deviation) or associated estimates of uncertainty (e.g. confidence intervals)
☒	☐	For null hypothesis testing, the test statistic (e.g. *F*, *t*, *r*) with confidence intervals, effect sizes, degrees of freedom and *P* value noted<br>*Give P values as exact values whenever suitable.*
☒	☐	For Bayesian analysis, information on the choice of priors and Markov chain Monte Carlo settings
☒	☐	For hierarchical and complex designs, identification of the appropriate level for tests and full reporting of outcomes
☐	☒	Estimates of effect sizes (e.g. Cohen's *d*, Pearson's *r*), indicating how they were calculated

Our web collection on statistics for biologists contains articles on many of the points above.

Software and code

Policy information about availability of computer code

Data collection	Data collection was by the commercial Xcalibur™ Software from Thermo Fisher Scientific.
Data analysis	Data analysis was by the commercial software Spectronaut (Biognosys AG, Schlieren, Switzerland), and our self built models. The models were implemented in Python (Anaconda distribution version 4.2.0, https://www.anaconda.com/) using Keras (version 2.2.4, https://keras.io/) with TensorFlow (version 1.11.0, https://www.tensorflow.org/) backend. Data preprocessing and visualization were conducted with R (version 3.5.1, https://www.r-project.org/). Source code was deposited on GitHub at https://github.com/lmsac/DeepDIA/.

For manuscripts utilizing custom algorithms or software that are central to the research but not yet described in published literature, software must be made available to editors/reviewers. We strongly encourage code deposition in a community repository (e.g. GitHub). See the Nature Research guidelines for submitting code & software for further information.

Data

Policy information about availability of data

All manuscripts must include a data availability statement. This statement should provide the following information, where applicable:

- Accession codes, unique identifiers, or web links for publicly available datasets
- A list of figures that have associated raw data
- A description of any restrictions on data availability

All raw mass spectrometry data, spectral libraries and search results are publicly available at the ProteomeXchange Consortium (http://proteomecentral.proteomexchange.org/). Raw data of HeLa, HEK-293, mouse and mixed proteome samples are available with the dataset identifier PXD005573 [http://proteomecentral.proteomexchange.org/cgi/GetDataset?ID=PXD005573], PXD006932 [http://proteomecentral.proteomexchange.org/cgi/GetDataset?ID=PXD006932], PXD004452 [http://proteomecentral.proteomexchange.org/cgi/GetDataset?ID=PXD004452], and PXD009875 [http://proteomecentral.proteomexchange.org/cgi/GetDataset?ID=PXD009875] (see Supplementary Table 1 for details). All the models for MS/MS, RT and detectability prediction, the data used for model training to generate PlasmaPredicted (see Supplementary Table 2 for details), raw data of serum samples, all in silico spectral libraries and the saved projects from Spectronaut have been deposited to ProteomeXchange via the iProX49 partner repository with the dataset identifier

PXD014108/IPX0001628000 [http://proteomecentral.proteomexchange.org/cgi/GetDataset?ID=PXD014108]. The source data underlying Fig. 2c-d and 3b-d, as well as Supplementary Fig. 1, 2, 3b-c, 4b-c, 5b-c, 7, 8c and 10b are provided as a Source Data file.

Field-specific reporting

Please select the one below that is the best fit for your research. If you are not sure, read the appropriate sections before making your selection.

- [x] Life sciences
- [ ] Behavioural & social sciences
- [ ] Ecological, evolutionary & environmental sciences

For a reference copy of the document with all sections, see nature.com/documents/nr-reporting-summary-flat.pdf

Life sciences study design

All studies must disclose on these points even when the disclosure is negative.

Sample size	The manuscript is focused on deep learning based bioinformatics for proteome data analysis. Most of the data used for models training were published by others, and include 3 technical replicates. More than 69,577 peptide spectra were used to train and test the models. For human serum sample analysis, three biological different serum samples were collected.
Data exclusions	There is no data exclusion.
Replication	There are 3 technical replicates to verify the quantitative results obtained by the deep learning based bioinformatic method to analyze data-independent acquisition proteomic data.
Randomization	Not applicable. The manuscript is about a deep learning based bioinformatics for proteome data analysis. We compare the results obtained by our algorithm and well recognized methods. And we spiked standard isotope labelled peptides into serum samples to verify our identification results.
Blinding	Not applicable. We describe a new method for proteome data analysis. The results were verified by comparison to the ones obtained with well recognized methods. We also spiked standard isotope labelled peptides into serum samples to verify our identification results.

Reporting for specific materials, systems and methods

We require information from authors about some types of materials, experimental systems and methods used in many studies. Here, indicate whether each material, system or method listed is relevant to your study. If you are not sure if a list item applies to your research, read the appropriate section before selecting a response.

Materials & experimental systems

n/a	Involved in the study	
[x]	[ ]	Antibodies
[x]	[ ]	Eukaryotic cell lines
[x]	[ ]	Palaeontology
[x]	[ ]	Animals and other organisms
[ ]	[x]	Human research participants
[x]	[ ]	Clinical data

Methods

n/a	Involved in the study	
[x]	[ ]	ChIP-seq
[x]	[ ]	Flow cytometry
[x]	[ ]	MRI-based neuroimaging

Human research participants

Policy information about studies involving human research participants

Population characteristics	Three healthy male volunteers at the ages of 20 to 40.
Recruitment	The volunteers were randomly recruited. We used their serum sample to verify our bioinformatic method, and we did not study any biological characters related proteomics.
Ethics oversight	Human serum samples were collected from volunteers under the consent of the donors. The protocol of blood collection, processing and MS analysis was approved by the Medical Ethics Committee of Shanghai Stomatological Hospital affiliated to Fudan University ([2016]0001), and complied with all relevant laws and regulations of China.

Note that full information on the approval of the study protocol must also be provided in the manuscript.
